# KmerSignificance score: A discriminative and biologically informed framework for viral *k*-mer prioritization

**DOI:** 10.1371/journal.pcbi.1014332

**Published:** 2026-10-27

**Authors:** Dylan Lebatteux, Florian Corso, Hugo Soudeyns, Isabelle Boucoiran, Soren Gantt, Abdoulaye Baniré Diallo

**Affiliations:** 1 Department of Computer Science, Université du Québec à Montréal, Montréal, Québec, Canada; 2 Centre de recherche Azrieli du CHU Sainte-Justine, Montréal, Québec, Canada; 3 Cité des Sciences et de l’Innovation de Guinée, Conakry, Guinea; 4 Department of Microbiology, Infectiology and Immunology, Faculty of Medicine, Université de Montréal, Montréal, Québec, Canada; 5 Department of Pediatrics, Faculty of Medicine, Université de Montréal, Montréal, Québec, Canada; 6 Pathology Advanced Translational Research Unit (PATRU), Department of Pathology and Laboratory Medicine, Emory University School of Medicine, Atlanta, Georgia, United States of America; 7 Department of Obstetrics and Gynecology, Faculty of Medicine, Université de Montréal, Montréal, Québec, Canada; Shenzhen University of Advanced Technology, CHINA

## Abstract

Distinguishing closely related viral strains requires identifying genomic regions where subtle sequence differences carry biological significance. While *k*-mer-based approaches offer computational efficiency for genome analysis, existing methods lack standardized frameworks for evaluating which *k*-mers are most informative. Current selection strategies focus primarily on statistical discriminative power without integrating biological context. We introduce KmerSignificance Score (KSS), a *k*-mer prioritization framework combining three components: an information-theoretic method measuring strain-distinguishing capacity, an optimized amino acid substitution matrix (MIYATA_EVO) for mutation impact assessment, and a protein characterization score derived from annotations and database cross-references retrieved through UniProt. All three component scores and the integrated KSS are bounded in [0,1] by construction, providing a common numerical scale for ranking positions within a dataset. The discriminative component achieved a mean F1 of 0.881, not significantly different from chi-squared (0.876), the strongest of eight baselines. Top-ranked selections from the discriminative component nonetheless differed from those produced by chi-squared, especially for SARS-CoV-2 and HIV-1. The MIYATA_EVO matrix, optimized using a genetic algorithm, increased the composite agreement score across eight amino acid characteristics by 28.4% over the original MIYATA matrix. Across 17,471 viral proteins, the protein characterization score tracked UniProt’s five-level annotation score closely (Kendall’s τ=0.775), as expected for measures drawing on overlapping annotation content. Within each level, the continuous score provided finer numerical resolution among proteins. Literature cross-referencing on SARS-CoV-2 (278,738 sequences, 19 Pango-derived lineage labels), HIV-1 (12,223 sequences, 15 subtype labels), and human cytomegalovirus (HCMV; 399–646 sequences, 4–8 locus-specific genotypes) found that the top-ranked *k*-mers reported here map to established variant-defining mutations, subtype-specific polymorphisms, and genotype markers. KSS provides a standardized framework for viral *k*-mer prioritization with potential applications in variant surveillance, molecular epidemiology, and functional annotation. The tool is available at https://github.com/bioinfoUQAM/KmerSignificanceScore.

## Author summary

Identifying genetic differences between closely related viral strains is essential for pandemic preparedness, vaccine development, and understanding disease outbreaks. With millions of viral genomes now sequenced, researchers need tools that can rapidly prioritize genomic differences for biological investigation, not just identify those that are statistically distinctive. Current methods based on short genomic sequences distinguish viral strains but generally do not incorporate biological information about the encoded proteins. We developed KmerSignificance Score (KSS), a framework that ranks genomic positions by combining three types of evidence: how well they distinguish viral strains, how much the encoded amino acid changes affect protein properties, and how thoroughly the affected protein has already been studied. We placed the resulting scores on a 0-to-1 scale, so that each position can be scored on its own rather than relative to the rest of the dataset. We applied our framework to three major human pathogens (SARS-CoV-2, HIV-1, and human cytomegalovirus) and found that the top-scoring positions reported here correspond to sites with documented roles in immune evasion, drug resistance, viral fitness, and strain classification. KSS can help prioritize genomic features for surveillance of emerging variants, guide experimental validation, and support molecular epidemiology.

## Introduction

High-throughput sequencing technologies have substantially expanded the global virome catalog. GenBank contained 6.8 million viral genomes by the end of 2021, and the database has continued to grow since then [1]. This extensive diversity presents significant computational challenges for viral characterization and surveillance [2,3]. Alignment-free approaches using *k*-mers (short nucleotide or amino acid subsequences) have emerged as efficient tools for capturing genetic signatures that distinguish viral lineages and strains [4–10].

While *k*-mer-based methods efficiently process large-scale genomic data, determining which *k*-mers merit biological investigation remains challenging. Existing selection strategies suffer from key limitations that reduce interpretability and cross-study applicability. Motif discovery tools such as MEME and STREME identify recurring or enriched sequence patterns but are not designed to produce position-wise scores for multi-class discrimination [11,12]. Traditional feature selection frameworks, including univariate filters (chi-squared, mutual information, ANOVA) and machine learning techniques (Random Forest, LASSO), generate dataset-specific score distributions [7,13–15]. While some metrics like Cramér’s V and normalized mutual information produce theoretically bounded [0,1] values, their empirical distributions vary substantially across datasets with different class structures. This complicates the interpretation of equal raw scores across viruses.

Most critically, current strategies excel at identifying statistically informative patterns but lack integrated mechanisms to evaluate biological significance. Recent approaches such as GRAMEP [16] illustrate this trend, achieving efficient entropy-based *k*-mer selection for viral variant identification without incorporating biological context into the selection score. This disconnect between computational pattern recognition and functional understanding limits our ability to prioritize *k*-mers associated with mutations affecting pathogen phenotypes such as host adaptation, immune escape, and pathogenicity [17]. To address these limitations, we developed KmerSignificance Score (KSS), a *k*-mer prioritization framework integrating three complementary components. First, a discriminative component quantifies strain-distinguishing capacity through information-theoretic analysis with adaptive complexity scaling. Second, an optimized amino acid substitution matrix (MIYATA_EVO) assesses the biophysical impact of each substitution. It was derived by optimizing MIYATA, which had the highest composite agreement score among the 37 candidates evaluated against eight amino acid characteristics. Third, a protein characterization score integrates annotations and database cross-references retrieved through UniProt to quantify how thoroughly each protein has been characterized. All three component scores and the integrated KSS are bounded in [0,1] by construction, without normalization across positions within a dataset. Each position can therefore be scored independently of the others on a common numerical scale.

We evaluated the three KSS components separately and assessed the integrated score across SARS-CoV-2, HIV-1, and human cytomegalovirus (HCMV), three viruses with distinct evolutionary rates, genome organizations, and classification frameworks. Aggregate mean F1 did not differ significantly between the discriminative component and chi-squared, the strongest of eight baselines, and the per-unit differences varied in sign. Top-ranked selections from the discriminative component differed from those produced by chi-squared, especially for SARS-CoV-2 and HIV-1. Position-level null distributions showed that chance thresholds differed across datasets, while the highest-scoring positions exceeded the highest threshold estimated in any dataset. Compared with the original MIYATA matrix [18], MIYATA_EVO improved agreement with seven of the eight characteristics evaluated. The protein characterization score followed the ordering of UniProt’s five-level annotation score while providing finer resolution among proteins within each category. Literature cross-referencing showed that the top-ranked *k*-mers reported here map to documented functional or strain-distinguishing loci across all three viruses. For SARS-CoV-2, top-ranked positions were also enriched in the most selective layers of an external mutation catalog, although no layer provides a complete genome-wide benchmark. In the implementations tested, the per-position scoring cost of the discriminative component stayed within the same order of magnitude as that of chi-squared, and the mutational and protein components added little marginal computation.

## Materials and methods

### Ethics Statement

This study exclusively used publicly available sequence data from GenBank and the Los Alamos National Laboratory HIV Sequence Database. No human subjects, animal experiments, or personally identifiable information were involved. No ethics approval was required.

### KmerSignificance score framework

KSS combines discriminative capacity with biological context through an integrated three-component system. The framework comprises: (1) a discriminative component quantifying strain-distinguishing capacity through information-theoretic analysis, (2) a mutational component assessing biophysical impact through an optimized amino acid substitution matrix (MIYATA_EVO), and (3) a protein characterization component, built from the annotations and database cross-references retrieved through UniProt. Each component score is bounded in [0,1] by construction.

Fig 1 illustrates the complete computational workflow comprising five main stages: input processing, coding sequence (CDS) translation and alignment, *k*-mer extraction with mutation identification, multi-component scoring, and structured output generation.

**Fig 1:**
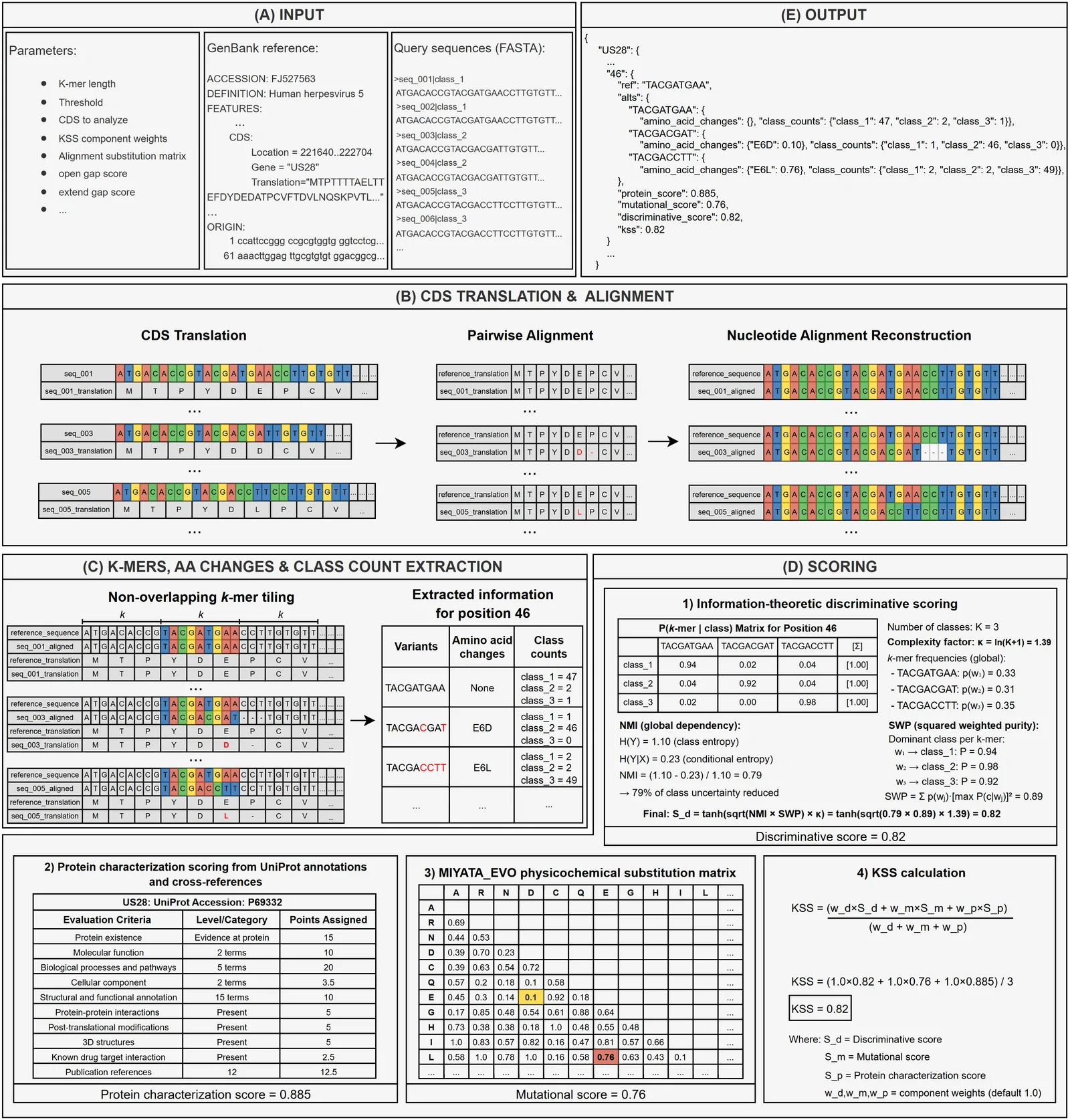
KSS computational workflow. **(A)** Input requirements: user-defined parameters (*k*-mer length, prevalence threshold *t*, component weights), GenBank reference genome with CDS annotations, and query sequences in FASTA format with class labels in the headers. **(B)** CDS translation and alignment: amino acid sequence generation, pairwise alignment against reference, and nucleotide alignment reconstruction guided by protein alignment. (**C**) *k*-mer extraction using non-overlapping tiling with amino acid change identification and class frequency tabulation for each position. **(D)** Multi-component scoring: (1) information-theoretic discriminative scoring, (2) protein characterization scoring, (3) MIYATA_EVO-based mutational impact scoring, and (4) weighted integration into KSS. **(E)** Structured JSON output with *k*-mer sequences, component scores and KSS values. The three-class example runs from panel A to panel **E.**

#### Data preprocessing and *k*-mer extraction.

KSS expects three inputs: in-frame viral coding sequences in FASTA format with user-supplied class labels, a GenBank reference genome, and user-defined settings. These settings specify the coding sequences to analyze, *k*-mer length (*k*), the prevalence threshold (*t*), the alignment substitution matrix, and the gap penalties. The prevalence threshold is the minimum proportion of sequences within at least one class that must contain a given *k*-mer. Default numerical settings are *k* = 9 nucleotides, shown to be effective for capturing discriminative viral signatures [6,7,19]; *t* = 0.25, retaining *k*-mers present in at least 25% of the sequences of at least one class; and Needleman-Wunsch [20] gap penalties of −10 for opening and −1 for extension.

The preprocessing pipeline comprises four sequential steps:

(1) **CDS translation:** Sequences are translated using the standard genetic code to generate amino acid sequences for alignment.
(2) **Pairwise alignment:** For each gene, query amino acid sequences are aligned, using the Needleman-Wunsch algorithm [20] with BLOSUM62 by default, against the longest translation among CDS features bearing the matching gene qualifier in the GenBank reference.
(3) **Nucleotide alignment reconstruction:** The amino acid alignment guides reconstruction: observed codons, including synonymous variants, are copied directly from the original nucleotide sequences, and amino acid gaps are represented by three-nucleotide gaps.
(4) **Variant identification and *k*-mer extraction:** Using adapted KANALYZER functions [21], we extract *k*-mers, variants, amino acid mutations, and class occurrence frequencies from aligned sequences, preparing data for multi-component scoring.

#### Discriminative component.

The discriminative component quantifies each position’s ability to distinguish among predefined classes within a single virus through information-theoretic analysis combined with *k*-mer purity assessment. This approach integrates normalized mutual information with class-specific *k*-mer enrichment, scaled by class complexity.

Let $𝒮=\{s_{1},...,s_{N}\}$ denote the sequence set and $𝒞=\{c_{1},...,c_{K}\}$ the *K* labels of the user-supplied partition of 𝒮, hereafter called classes, with label $y_{i}\in 𝒞$ for each sequence. For a given position, let $w_{1},...,w_{n}$ denote the *k*-mers retained at the prevalence threshold *t*, which include the reference *k*-mer when it meets that threshold, and let $X\in \{0,1{\}}^{N\times n}$ be the binary feature matrix where $X_{ij}=1$ if $w_{j}$ is observed in sequence $s_{i}$. A sequence whose *k*-mer at that position does not meet the threshold contribu*t*es an all-zero row.

We first compute the entropy of the class distribution:


$$\begin{array}{c} H(Y)=-\sum_{i=1}^{K}p(c_{i})\ln p(c_{i}) \end{array}$$ (1)

where $p(c_{i})=|{𝒮}_{i}|/N$ is the proportion of sequences belonging to class $c_{i}$.

The conditional entropy *H*(*Y*|*X*) captures the remaining uncertainty about class membership given the *k*-mer configuration:


$$\begin{array}{c} H(Y|X)=\sum_{𝐱\in 𝒳}p(𝐱)\;H(Y|X=𝐱) \end{array}$$ (2)

where 𝒳 denotes the set of unique *k*-mer configurations observed across sequences, *p*(**x**) is the proportion of sequences exhibiting configuration **x**, and *H*(*Y*|*X* = **x**) is the class entropy among sequences sharing that configuration.

The normalized mutual information quantifies the proportional reduction in class uncertainty:


$$\begin{array}{c} \text{NMI}=\frac{H(Y)-H(Y|X)}{H(Y)} \end{array}$$ (3)

This asymmetric normalization quantifies the fraction of class uncertainty resolved by the *k*-mer configuration, which is appropriate for feature selection where predictive capacity toward the target variable is of primary interest.

To capture class-specific *k*-mer enrichment, we define the purity of each *k*-mer variant as $\pi_{j}=\max_{i}P(c_{i}|w_{j})$, representing the dominant class proportion among sequences containing variant $w_{j}$. The squared weighted purity across all variants at the position is then:


$$\begin{array}{c} \text{SWP}=\sum_{j=1}^{n}p(w_{j})\cdot \pi_{j}^{2} \end{array}$$ (4)

where $p(w_{j})$ is the frequency of *k*-mer variant $w_{j}$ across all sequences. Squaring the purity term increases the contrast within SWP between highly pure *k*-mers, strongly associated with a single class, and moderately pure ones. The prevalence threshold *t* keeps isolated variants out of this term, since a variant carried by a single sequence is perfectly pure by cons*t*ruction.

To account for the number of classes in the scoring function, we introduce an adaptive complexity parameter:


$$\begin{array}{c} \kappa =\ln(K+1) \end{array}$$ (5)

At fixed NMI and SWP, this logarithmic scaling raises the score as the number of classes grows, so that a given association counts for more when it separates many classes than when it separates two. Table 1 gives the values for different class numbers.

**Table 1:** Adaptive complexity parameter κ as a function of the number of classes *K.*

*K*	κ
2	1.10
3	1.39
5	1.79
10	2.40
20	3.04

The final discriminative score is:


$$\begin{array}{c} S_{d}=\tanh\left(\sqrt{\text{NMI}\times \text{SWP}}\times \kappa \right) \end{array}$$ (6)

The geometric mean combines the information-theoretic and purity terms, κ grows logarithmically with the number of classes, and the hyperbolic tangent bounds the score in [0,1] without requiring global dataset normalization. Values approaching 1 indicate positions where *k*-mer patterns strongly predict class membership, while values near 0 correspond to minimal class-distinguishing capacity.

#### Mutational component.

Amino acid substitutions influence protein function through alterations in biophysical properties such as size, charge, and polarity [22]. Substitutions between similar residues typically maintain structural stability, while changes involving residues with divergent properties often disrupt function [22–24].

Substitution matrices quantitatively assess these effects by correlating scores with physicochemical properties [23,25]. While traditional matrices (PAM, BLOSUM) derive from evolutionary frequencies [26,27], we employ MIYATA_EVO, an optimized physicochemical matrix that directly quantifies biophysical dissimilarity. MIYATA_EVO was derived by applying genetic algorithm optimization to the original MIYATA matrix [18], maximizing the sum of correlations between the mutational impact scores derived from the matrix and the eight amino acid characteristics described in the following section.

The matrix undergoes two preprocessing steps. First, distance values are inverted so that biochemically similar amino acid pairs receive high values:


$$\begin{array}{c} M^{\prime }(a,b)=M_{\max}-M(a,b) \end{array}$$ (7)

Second, values are normalized to [0,1] using min-max scaling:


$$\begin{array}{c} M^{\prime \prime }(a,b)=\frac{M^{\prime }(a,b)-M_{\min}^{\prime }}{M_{\max}^{\prime }-M_{\min}^{\prime }} \end{array}$$ (8)

After these transformations, similar amino acid pairs have values approaching 1, while dissimilar pairs approach 0.

For each substitution, the mutational impact is computed as:


$$\begin{array}{c} S_{m}=\max(1-M^{\prime \prime }(a_{i},b_{i}),\;0.1) \end{array}$$ (9)

where $a_{i}$ and $b_{i}$ represent the original and mutated amino acids. This inversion converts similarity into impact: dissimilar pairs (low $M^{\prime \prime }$) yield high impact scores, while similar pairs (high $M^{\prime \prime }$) produce low scores. The score is computed from recorded amino acid changes, so synonymous substitutions produce no entry and therefore contribute zero. The floor of 0.1 keeps every non-synonymous substitution above zero, including Leu↔Met and Met↔Val, the two pairs the MIYATA_EVO optimization drove to zero distance. It sits in the gap the unfloored impact scores leave between 0.079 and 0.106, so it affects the 8 closest of 190 amino acid pairs and leaves every larger matrix-derived impact untouched. Its largest correction changes the final KSS by at most 0.033 under equal component weights.

For positions with multiple substitutions, $S_{m}$ is their maximum impact score, so that the highest-scoring substitution is not diluted by averaging. Insertions and deletions are assigned a configurable score in [0,1], 1.0 by default.

#### MIYATA_EVO matrix optimization.

The mutational component was evaluated against eight amino acid characteristics using 37 candidate matrices and rate models: 30 published matrices whose values were matched to Biopython, the AAindex database [28], and/or the primary literature, VTML200 [29], WAG [30], and five rate models estimated from viral alignments (HIVb and HIVw [31], rtREV [32], FLU [33] and FLAVI [34]). S1 Table gives the complete inventory, the sources, and the classification into five families. MIYATA_EVO, the product of the optimization described below, is reported separately from the candidate panel. Six candidates were published as rate models: WAG and the five viral models. For each, the rate matrix *Q* was normalized to one expected substitution per site, P(δ)=exp(Qδ) was evaluated at a conversion divergence δ, and integer third-bit log-odds scores were obtained as $S_{ij}=round[(3/\ln2)\ln(P_{ij}(\delta )/\pi_{j})]$. We used δ=2.5, comparable with the 250-PAM matrices already in the panel, and assessed sensitivity over δ∈{2,2.5,3,4} (S1 Table).

The eight characteristics comprise seven biophysical properties (Table 2): hydrophobicity, polarity, side chain volume, accessible surface area, isoelectric point, interior-exterior transfer propensity, and aromaphilicity, plus minimum codon distance (MinCodD) as a genetic substitution measure.

**Table 2:** Amino acid biophysical properties for substitution matrix optimization.

AA	Hydrophob.	Polarity	Volume	Area Lost	pI	In-Out	Aroma.
Ala	0.31	8.10	27.5	0.74	6.00	0.20	0.025
Arg	-1.01	10.50	105.0	0.64	10.76	-1.34	0.75
Asn	-0.60	11.60	58.7	0.63	5.41	-0.69	0.20
Asp	-0.77	13.00	40.0	0.62	2.77	-0.72	-0.025
Cys	1.54	5.50	44.6	0.91	5.05	0.67	0.20
Gln	-0.22	10.50	80.7	0.62	5.65	-0.74	0.30
Glu	-0.64	12.30	62.0	0.62	3.22	-1.09	0.05
Gly	0.00	9.00	53.2	0.72	5.97	0.06	0.00
His	0.13	10.40	79.0	0.78	7.59	0.04	0.45
Ile	1.80	5.20	93.5	0.88	6.02	0.74	0.20
Leu	1.70	4.90	93.5	0.85	5.98	0.65	0.125
Lys	-0.99	11.30	100.0	0.52	9.74	-2.00	0.10
Met	1.23	5.70	94.1	0.85	5.74	0.71	0.325
Phe	1.79	5.20	115.5	0.88	5.48	0.67	0.575
Pro	0.72	8.00	108.2	0.64	6.30	-0.44	0.125
Ser	-0.04	9.20	29.3	0.66	5.68	-0.34	0.125
Thr	0.26	8.60	51.3	0.70	5.66	-0.26	0.075
Trp	2.25	5.40	145.5	0.85	5.89	0.45	1.00
Tyr	0.96	6.20	117.3	0.76	5.66	-0.22	0.85
Val	1.22	5.90	71.5	0.86	5.96	0.61	0.15

**AA:** Amino acid three-letter code. **Property definitions: Hydrophob.:** Hydrophobicity [35]. **Polarity:** Polarity index [36]. **Volume:** Side chain volume [37]. **Area Lost:** Accessible surface area fraction lost upon folding [38]. **pI:** Isoelectric point [39]. **In-Out:** Interior-exterior transfer propensity [40]. **Aroma.:** Aromaphilicity index [41]. An eighth characteristic, minimum codon distance (MinCodD), was also used for matrix optimization. Because it is defined for pairs of amino acids rather than for individual residues, it has no per-residue value to list here and is reported with the matrix-optimization results [42].

For each matrix, the mutational impact scores the pipeline derives were computed for all 190 unique amino acid pairs (20×19/2). Biophysical distances were calculated as absolute differences between property values for each amino acid pair. Spearman rank correlations between impact scores and biophysical distances were computed for the seven properties, and between impact scores and MinCodD. The eight correlations were summed with equal weight to form a composite agreement score. The pairs are not independent, since each amino acid enters 19 of them, and the composite is also the objective the optimization below maximizes, so the correlations and the composite are reported as descriptive measures of agreement with the intended construct rather than as hypothesis tests.

The candidate matrix with the highest composite value (MIYATA [18]) was further optimized by a genetic algorithm against that same composite. Each individual encoded the 190 upper-triangle pairwise distances, which enforces symmetry by construction and fixes self-substitution distances to zero. With probability 0.5, an offspring was generated by uniform crossover of two survivors drawn at random, half of the pairwise distances coming from each parent, followed by fixed-step mutation. Otherwise it was generated by mutating a single survivor. Mutation perturbed 10% of the pairwise distances by ±0.1 and clipped them to [0, 10]. The algorithm used truncation selection (top 50%) with elitism preserving the best individual. The initial population of 1,000 individuals was generated by mutating the original MIYATA matrix, with the original retained as the initial elite. The algorithm ran for a maximum of 10,000 generations with early stopping after 100 consecutive generations without improvement (threshold 10^–6^).

#### Protein characterization score.

For each candidate position, the protein characterization component quantifies how thoroughly the protein encoded by the CDS containing that position has been characterized. It combines ten annotation categories recorded in that protein’s UniProt entry: Gene Ontology molecular function, biological process and cellular component terms; protein existence evidence; structural and functional annotations; protein-protein interactions; post-translational modifications; 3D structures; drug target cross-references; and literature references [43]. S2 Table gives the scoring thresholds, the fixed category weights, and the UniProt field or cross-reference each category is read from. All annotations and database cross-references are taken from that entry, without independently querying the referenced databases.

The fixed category weights define a maximum of 100 points and were chosen from domain knowledge rather than optimized. Individual category scores are summed and scaled to [0,1]:


$$\begin{array}{c} S_{p}=\frac{1}{100}\sum_{i=1}^{10}s_{i} \end{array}$$ (10)

Because $S_{p}$ is constant within a protein, it can change relative priorities across proteins but not the ordering of positions within the same protein.

#### KSS calculation.

KSS integrates the three component scores through weighted averaging:


$$\begin{array}{c} \text{KSS}=\frac{w_{d}\cdot S_{d}+w_{m}\cdot S_{m}+w_{p}\cdot S_{p}}{w_{d}+w_{m}+w_{p}} \end{array}$$ (11)

where $S_{d}$, $S_{m}$, and $S_{p}$ are the discriminative, mutational and protein characterization scores, and $w_{d}$, $w_{m}$, $w_{p}$ are user-defined non-negative weights, not all zero.

The translated reference coding sequence defines a grid of non-overlapping, reference-anchored nucleotide windows, and each KSS value in the integrated output corresponds to one window of that grid. Windows at which no sequence differs from the reference are not emitted. Let *L* be the nucleotide length of the translated reference coding sequence, excluding the terminal stop codon, and let the *k*-mer length be a multiple of 3, which the default of 9 nucleotides satisfies. For


$$\begin{array}{c} r\in \left\{0,...,\left\lfloor \frac{L}{k}\right\rfloor -1\right\},\;j=\frac{rk}{3},\;W_{j}=[3j,\;3j+k),\;A_{j}=\left\{j,...,j+\frac{k}{3}-1\right\} \end{array}$$ (12)

*j* is the zero-based codon index at which the window begins, $W_{j}$ is its interval of reference nucleotides, and $A_{j}$ is the corresponding set of reference amino acids, three of them at the default *k* = 9. Any terminal remainder containing fewer than *k* reference nucleotides lies outside this grid. A window is also omitted when an alignment leaves it with fewer than *k* reference nucleotides. Insertions preceding a window shift its origin in the alignment. If *q* inserted codons fall inside it, the aligned slice is extended to $k^{\prime }=k+3q$ nucleotides, covering $k^{\prime }/3$ aligned amino acid columns while retaining *k*/3 reference codons. The reading frame and the reference coordinates are preserved throughout.

The three components are defined at different biological levels and enter this common position as follows. $S_{d}$ is computed from the distinct nucleotide strings observed at $W_{j}$ across classes. $S_{m}$ is the maximum impact among the amino acid changes in the aligned slice associated with $W_{j}$, inserted residues included, which reduces them to a single value for the window. $S_{p}$ is the characterization score of the encoded protein and is therefore constant across all windows of that gene.

Because the component scores are bounded in [0,1] and the weights are non-negative, KSS also lies in [0,1]. The default $w_{d}=w_{m}=w_{p}=1$ gives equal weight to the three components, and setting a weight to zero removes that component. Component scores are rounded to three decimals before averaging, and the resulting KSS is rounded likewise.

### Validation and benchmarking

#### Viral datasets.

We selected three viruses with complementary characteristics for KSS validation. SARS-CoV-2 represents rapidly evolving positive-sense RNA viruses with extensive genomic surveillance data and well-defined variant classifications. HIV-1, a highly diverse retrovirus with complex subtype structure, tests robustness across extreme sequence variation. HCMV represents a DNA virus with moderate evolutionary rates and established genotype-defining mutations enabling targeted validation. Sequences were collected in November 2025 from GenBank [1] (SARS-CoV-2, HCMV) and the Los Alamos National Laboratory HIV Sequence Database [44] (HIV-1).

The collected sequences were filtered using four quality control criteria: (1) exclusion of unverified sequences, (2) nucleotide sequence length within ±10% of the translated reference coding sequence, (3) complete coding sequences without premature stop codons, and (4) less than 1% ambiguous (non-ACGT) nucleotides. Classes were retained when at least 20 sequences remained after filtering, and all qualifying sequences in those classes were included without subsampling.

The genes analyzed were ORF1ab, S, M, N, and E for SARS-CoV-2; gag, pol, and env for HIV-1; UL55, UL73, and US28 for HCMV. Class assignments followed three independently established schemes: SARS-CoV-2 labels are Pango lineage designations [45] carried by the NCBI Virus records, and the analyzed lineages were restricted to those represented in the Nextclade reference tree [46], which also places B.1.427 and B.1.429 in one clade, 21C, so they are analyzed under a single label; HIV-1 labels come from the subtype field of the Los Alamos HIV Sequence Database [44] and comprise phylogenetically defined group M subtypes, circulating recombinant forms identified from shared mosaic genome structures, and group O; HCMV labels are locus-specific genotypes defined from amino acid polymorphism profiles [47–49], which is why their number varies between genes. The three HCMV locus-specific datasets were derived from overlapping genome accessions: 325 accessions were represented at all three loci, among 791 unique accessions across UL55, UL73, and US28. Each locus was nevertheless analyzed as a separate classification task, with its own gene sequences, genotype labels, train-test splits, and fitted models. S3 Table gives the complete inventory. Reference genomes for mutation reporting were NC_045512 (SARS-CoV-2), AF033819 (HIV-1), FJ616285 (UL55), and FJ527563 (US28, UL73). The analyses used *t* = 0.25 for SARS-CoV-2 and HCMV and *t* = 0.33 for HIV-1, where the higher value was a dataset-specific preprocessing choice motivated by extensive intra-subtype length polymorphism, not an established requirement. Sensitivity to *t* was evaluated from 0.10 to 0.50 for all three datasets over the same set of scored positions. At each threshold, we recorded the variance of the KSS distribution, the overlap with the selection made at the analysis threshold, and the Spearman correlation of the complete score vector (S4 Table). Dataset characteristics are summarized in Table 3.

**Table 3:** Viral genome datasets used for KSS validation.

Virus	Gene	Sequences	Classes	Length (nt)	Non-ACGT (%)
**SARS-CoV-2**	ORF1ab S M N E	278,738	19 [113–101,757]	21,282 ± 3 3,814 ± 4 669 ± 0 1,256 ± 4 228 ± 0	0.019 0.018 0.003 0.003 0.000
**HIV-1**	gag pol env	12,223	15 [27–6,850]	1,502 ± 15 3,013 ± 22 2,577 ± 29	0.031 0.027 0.047
**HCMV**	UL55 UL73 US28	399 646 443	4 [39-196] 8 [47-112] 6 [29-162]	2,722 ± 3 412 ± 4 1,065 ± 1	0.000 0.001 0.000

**Data sources:** SARS-CoV-2 and HCMV sequences from GenBank; HIV-1 sequences from Los Alamos National Laboratory HIV Sequence Database. **Sequences:** Total number of sequences after quality filtering. For SARS-CoV-2 and HIV-1, the same multi-gene dataset is used across all genes. For HCMV, each locus was analyzed as a separate classification task, although the three locus-specific datasets share many of the same genome accessions. **Classes:** Number of distinct classes [minimum-maximum sequences per class]. SARS-CoV-2 labels follow the curated Pango nomenclature; HIV-1 labels are taken from the Los Alamos HIV Sequence Database subtype field; HCMV labels are locus-specific genotypes based on amino acid polymorphism profiles. **Length:** Mean nucleotide sequence length ± standard deviation. **Non-ACGT:** Percentage of ambiguous nucleotides or gap characters, indicating sequence quality and completeness. **Collection periods:** SARS-CoV-2 (2020–2025), HIV-1 (1978–2025), HCMV (2003–2025).

#### Discriminative component validation.

The discriminative component was benchmarked against seven established univariate feature selection methods: chi-squared test [50], mutual information (MI) and normalized mutual information (NMI) [51], odds ratio [52], Cramér’s V [53], the ANOVA F-statistic [54] and the normalized symmetric chi-squared divergence [55], along with a random baseline. The NMI baseline uses the symmetric arithmetic-mean normalization implemented in scikit-learn, which differs from the asymmetric normalization of Eq (3). The normalized symmetric chi-squared divergence is the normalized triangular discrimination of Topsøe [55], a non-metric divergence defined for two class-conditional *k*-mer relative-frequency profiles *p* and *q* as $d(p,q)=\frac{1}{2}\sum_{i}(p_{i}-q_{i}{)}^{2}/(p_{i}+q_{i})$, computed at each position for every pair of classes with non-empty profiles at that position and averaged over those pairs.

**Classification benchmark.** Evaluation employed linear Support Vector Machines (SVM), widely used in *k*-mer-based viral classification studies [6,7,56,57], with *C* = 1, a limit of 10,000 iterations, and the seed set to 42 + *i* at iteration *i*. Each method selected the top-*m* highest-scoring positions, with m∈{1,2,3,5,7,10,15,20,25}, pooled across genes for SARS-CoV-2 and HIV-1, whose genes share one class structure, and separately for UL55, UL73 and US28 in HCMV, whose genotyping schemes are locus-specific. We performed 100 iterations with stratified train-test splits, sized by adaptive stratified sampling at a target ratio of 70% constrained between 20 and 500 training sequences per class, the remaining sequences being used for testing. Within an iteration the prevalence filter and all selection scores were computed from the training sequences only, and the nine methods shared the resulting candidate *k*-mers, the split and the classifier seed. Comparisons between methods are exactly paired. Performance was measured by the macro-averaged F1 score and averaged over the five evaluation units, each defined by a common class structure (pooled SARS-CoV-2, pooled HIV-1, and HCMV UL55, UL73 and US28), and the nine values of *m*.

**Statistical analysis.** Paired differences were assessed with the corrected resampled *t*-test of Nadeau and Bengio [58], which replaces the variance factor 1/*n* by $1/n+n_{\text{test}}/n_{\text{train}}$ to account for the overlap between resampled training sets. In their notation the correlation surrogate is $\rho_{0}=n_{\text{test}}/(n_{\text{train}}+n_{\text{test}})$, and the quantity entering the corrected variance is $\rho_{0}/(1-\rho_{0})=n_{\text{test}}/n_{\text{train}}$, so that term is not itself a correlation. The cap of 500 training sequences per class makes this ratio differ between evaluation units, from 0.44 for the HCMV genes to 32.0 for SARS-CoV-2, the latter corresponding to $\rho_{0}=0.970$, and each unit was corrected with the ratio its own split produced. The global contrast is an unweighted mean over the *U* = 5 units. Treating the units as independent, its variance is $\mathrm{Var}(\bar{\Delta })=U^{-2}\sum_{u}v_{u}$, where $v_{u}=s_{u}^{2}(1/n+\rho_{u})$ and $\rho_{u}=n_{\text{test},u}/n_{\text{train},u}$. Its degrees of freedom were estimated using the Welch-Satterthwaite approximation, those of the per-unit contrasts being one fewer than the iteration count. Pointwise confidence intervals use the same corrected standard error and are not adjusted for multiplicity, while inferential claims use Holm-Bonferroni-adjusted *p*-values across the eight baseline comparisons [59]. Equivalence was assessed by two one-sided tests (TOST) [60], which establish equivalence only when the corrected 90% confidence interval lies entirely within the chosen bounds. We treated 0.01 macro-F1 units, one percentage point on the bounded F1 scale, as the largest difference we would regard as practically negligible for downstream classification, and examined 0.02 as a less stringent sensitivity margin, reported in S5 Table. The nine methods were also summarized descriptively by mean ranks over the 45 blocks defined by each evaluation unit crossed with each value of *m*, following Demšar [61] and using performance averaged over the 100 iterations. Because the nine values of *m* within a unit are nested rather than independent, no Friedman statistic or *p*-value is computed from them.

**Selection agreement.** Selection agreement was measured for each evaluation unit and each value of *m* by the Jaccard index between the top-*m* positions retained by the discriminative component and by each of the seven deterministic baselines, with ties broken by ascending position label, matching the classification benchmark, together with Spearman correlations between the complete score vectors. Both were computed once from scores obtained on the complete sequence set, rather than from the training-set scores generated within each resampling iteration, so they describe the full-data rankings produced by each criterion and do not quantify variation in selection overlap across benchmark splits.

**Cross-dataset comparability.** Cross-dataset comparability of the discriminative score was assessed against a position-level null. The score is a deterministic function of the *k*-mer-by-class contingency table, so permuting the class labels at fixed margins draws from the conditional null of that position without re-reading sequences or retraining a classifier. We drew 1,000 permutations per position and took the 95th percentile of the resulting distribution as that position’s null threshold, estimated by Monte Carlo. Permutation treats sequences as exchangeable, which sequences sharing a phylogeny are not: related sequences can share *k*-mers through common ancestry, making the permutation null too narrow. The calculation was therefore repeated with both margins scaled to a half, a quarter and a tenth of their nominal size, each reduction widening the null. Those reductions are a stress test spanning an order of magnitude, not an estimate of an effective sample size, which would require a tree or explicitly defined dependence blocks.

#### Protein-component calibration against UniProt annotation scores.

The protein component was compared with UniProt annotation scores, a five-level heuristic indicator of the annotation content of an entry (GO terms, functional comments and sequence features among others), giving greater weight to experimentally supported than to predicted annotations. The analysis described concordance and systematic differences between two scoring frameworks that are computed differently but draw on overlapping annotation content from the same UniProt entries.

Analysis used the 17,471 reviewed viral proteins of UniProtKB/Swiss-Prot (release 2025_02, taxonomy ID 10239, retrieved 1 June 2025) obtained via REST API in JSON format. Spearman rank correlation and Kendall’s tau coefficients evaluated the relationship between the UniProt annotation score and $S_{p}$. Confidence intervals (95%) were calculated using Fisher z-transformation and standard error approximation, respectively.

Proteins were stratified by UniProt annotation score (1–5), and variation in $S_{p}$ within each category was summarized using the mean, standard deviation, range and coefficient of variation. No null-hypothesis test was performed.

Discordance analysis identified proteins on which the two readings of the same records disagree substantially. UniProt annotation scores were normalized to [0,1] using (s−1)/4 where *s* is the original score. Discordance was quantified as:


$$\begin{array}{c} \Delta =S_{p}-{\text{UniProt}}_{\text{normalized}} \end{array}$$ (13)

Proteins with |Δ|>0.30 were identified as discordant. The threshold is descriptive, chosen to isolate moderate-to-large discrepancies rather than calibrated against any error rate. Cases with Δ>0.30 (high $S_{p}$ relative to UniProt) and Δ<−0.30 (low $S_{p}$ relative to UniProt) were analyzed separately. Because both scores are discrete, many differences equal the threshold exactly and were excluded by applying the strict inequality in decimal arithmetic.

#### Functional and discriminative site validation.

The integrated KSS framework was evaluated by comparing top-ranking genomic positions with literature-documented functional or strain-distinguishing loci across the three viruses. A position was counted as concordant if it matched either a documented functional locus or a strain-distinguishing locus. Support from both was not required.

**Concordance with published sites.** For SARS-CoV-2 and HIV-1, the top 12 genome-wide positions ranked by KSS were compared against documented functional and strain-defining sites from published literature. To prevent overrepresentation of indel-driven positions, which receive a uniform mutational score of 1.0 and can dominate rankings in highly diverse viruses such as HIV-1, a maximum of three indel positions was permitted within each top 12 selection, ensuring that substitution-based positions, scored by the optimized MIYATA_EVO matrix, constitute the majority of each selection. For HCMV, gene-specific analysis identified the top 10 positions per gene (UL55, UL73, US28) with the same indel constraint, because the three genes carry separate class assignments and are compared with gene-specific literature [47–49]. Positions with identical KSS values were ordered by gene name and then by ascending nucleotide position. To assess the contribution of the protein component, rankings were recomputed with $w_{p}=0$ and $w_{d}=w_{m}=1$, then compared with the default-weight rankings before and after the three-indel selection rule. Literature sources were identified through targeted searches combining viral species, gene names, and specific mutations identified by KSS. Concordance was recorded at the exact amino acid position where a site-specific annotation exists for that residue. Where the literature documents a structural or functional region rather than a residue, the position is reported as localizing to that region, and regional membership is not treated as an exact site match.

**Reference-specific error rates.** For SARS-CoV-2, we additionally computed error rates against COV2Var annotation layers [62] within the five analyzed genes. We admitted layers that annotate a mutation-level functional consequence and rest on a measurement or on a statistical test over observed sequences, together with membership in the catalog itself, which is reported for coverage rather than as functional evidence. Prediction layers, whose values are model output rather than observation, and host or patient metadata layers, which annotate a sequence rather than a position, are listed in S6 Table but not used as empirical validation. For each admitted layer and each selection size m∈{10,12,25,50,75,100} that does not exceed half of its evaluable universe, confusion counts were computed on the unfiltered pooled SARS-CoV-2 ranking, without the three-indel selection rule applied in the literature comparison. A selected *k*-mer absent from the layer is a false positive, and an annotated *k*-mer not selected is a false negative, giving FP/(FP + TN) and FN/(TP + FN). Precision and false discovery rate are reported separately. Coverage defines the evaluable universe: layers assaying a restricted region are evaluated only on *k*-mers carrying an assayed residue, and substitution-only layers exclude insertions and deletions. Chance expectations were computed both from the evaluable universe and after matching the gene composition of the selection, that universe being mostly ORF1ab while the top of the ranking is mostly spike. Enrichment over the evaluable universe was tested with the exact hypergeometric distribution, and the ranking of each layer with a one-sided Mann-Whitney rank-sum test, whose normalized statistic is the area under the receiver operating characteristic curve (AUROC) reported beside it. Every eligible layer is reported at every selection size its universe admits, so neither was chosen after seeing a result, and the probabilities are reported as nominal.

#### Computational cost assessment.

Analytical complexity was assessed per position in terms of the number of sequences *N*, the number of distinct *k*-mers *V* at that position, and the number of classes *K*. Per-position scoring times were measured on all eleven analyzed genes, comparing the discriminative component with chi-squared and mutual information on the same position-indexed matrix. Each scorer was run once as a warm-up and then timed three times over all scored positions of a gene, and the median elapsed time was divided by the number of positions. The split between preprocessing and scoring was measured on SARS-CoV-2 E and HCMV US28, with a single worker, so that local scoring cost is separated from the speed-up that distributing the pairwise alignments provides. The once-per-gene UniProt retrieval is excluded from both. All timings were taken on one machine, an Intel Core i9-14900HX with 64 GB of RAM.

## Results and discussion

### Discriminative component validation

#### Classification performance.

The discriminative component achieved a mean F1 of 0.881 across the five evaluation units and the nine values of *m*, against 0.876 for chi-squared, 0.866 for odds ratio, 0.862 for NMI, 0.858 for MI, 0.846 for the normalized symmetric chi-squared divergence, 0.780 for ANOVA, 0.706 for Cramér’s V and 0.555 for the random baseline (Fig 2).

**Fig 2:**
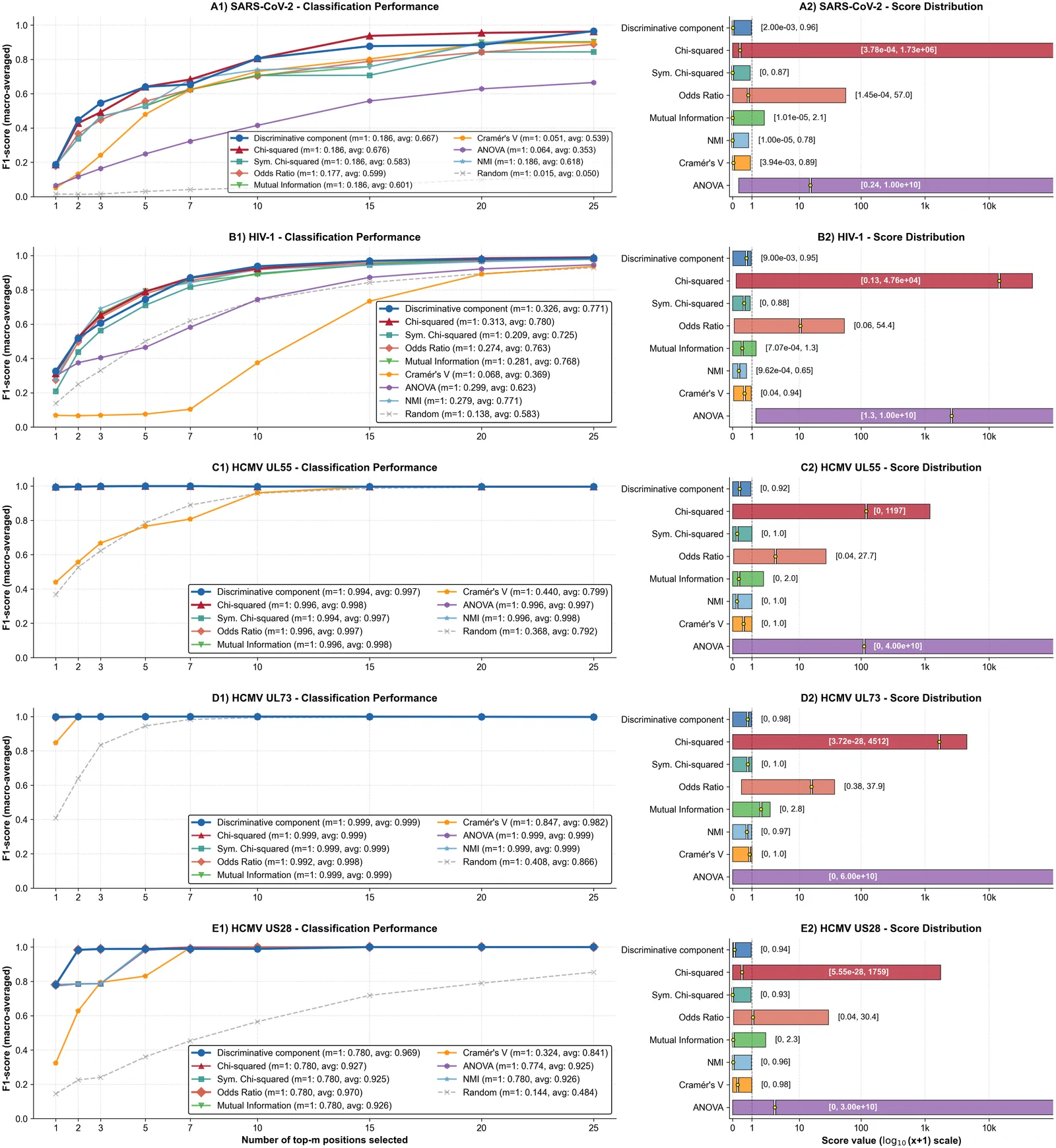
Discriminative component validation across viruses. Left panels: classification performance (macro-averaged F1) against the number of top-*m* selected positions, for nine feature selection methods. Right panels: empirical score ranges on a common $\log_{10}(x+1)$ axis, for the eight deterministic methods, over the benchmark universe. Discriminative component scores (blue) are bounded within [0,1]; baseline scores keep their native scales. Bars span the observed range, the marker gives the median, the dashed line marks a score of one, and bars past the truncated axis are cut at the frame. NMI: normalized mutual information; Sym. chi-squared: normalized symmetric chi-squared divergence. Legend entries give performance at *m* = 1 and the mean over all *m*, from 100 stratified iterations per unit. Interval estimates: Table 4, per unit S5 Table.

Paired analysis over the 100 iterations places these differences on an interpretable scale (Table 4). The discriminative component significantly outperformed three of the seven established baselines, Cramér’s V (+ 0.175, [+ 0.063, + 0.287]), MI (+ 0.022, [+ 0.010, + 0.035]) and NMI (+ 0.019, [+ 0.006, + 0.031]), as well as the random baseline (ΔF1 = + 0.326, 95% CI [+ 0.277, + 0.374]), all with Holm-adjusted *p* < 0.05. After Holm adjustment, four comparisons were inconclusive: the normalized symmetric chi-squared divergence (+ 0.035, [+ 0.004, + 0.066], *p* = 0.102), ANOVA (+ 0.101, [−0.014,+0.216], *p* = 0.255), odds ratio (+ 0.015, [−0.111,+0.142], *p* = 1.000) and chi-squared itself (+ 0.005, [−0.013,+0.023], *p* = 1.000).

**Table 4:** Paired comparison of the discriminative component with the eight baselines.

Method	Mean rank	Mean F1	ΔF1	95% CI	*p* (Holm)	Equivalent
Chi-squared	2.98	0.876	+ 0.005	[−0.013, + 0.023]	1.000	no
**Discriminative component**	3.38	0.881	reference method			
NMI	3.97	0.862	+ 0.019	[+ 0.006, + 0.031]	0.019	no
MI	4.13	0.858	+ 0.022	[+ 0.010, + 0.035]	0.003	no
Odds ratio	4.76	0.866	+ 0.015	[−0.111, + 0.142]	1.000	no
Symmetric chi-squared	5.01	0.846	+ 0.035	[+ 0.004, + 0.066]	0.102	no
ANOVA	5.61	0.780	+ 0.101	[−0.014, + 0.216]	0.255	no
Cramér’s V	6.59	0.706	+ 0.175	[+ 0.063, + 0.287]	0.015	no
Random baseline	8.58	0.555	+ 0.326	[+ 0.277, + 0.374]	< 10^–26^	no

**Method:** NMI, normalized mutual information; MI, mutual information; Symmetric chi-squared, normalized symmetric chi-squared divergence; ANOVA, ANOVA F-statistic. **Mean rank:** Rows are ordered by this column. Within each of the 45 blocks defined by evaluation unit and selection size, methods were ranked by mean F1 (1 = highest), and ranks were averaged across blocks. Because ranks summarize within-block ordering rather than the magnitude of F1 differences, their ordering need not match that of overall mean F1. Nothing inferential is computed from them. Δ**F1:** Mean paired difference in macro-averaged F1, discriminative component minus baseline, over the 100 iterations after averaging the five evaluation units within each iteration. **95% CI:** Interval unadjusted for multiplicity, based on the corrected resampled standard error [58], each unit contributing the correction its own split produced and the global variance combining the five, with Welch-Satterthwaite degrees of freedom. ***p* (Holm):** Corrected resampled *t*-test adjusted across *t*he eight baseline comparisons. **Equivalent:** Equivalence to the discriminative component established by two one-sided tests at a margin of 0.01 F1 units. Per-unit outcomes are given in S5 Table.

Against chi-squared, the highest-performing established baseline, the difference was not statistically significant after correction, and equivalence within the margin of 0.01 F1 units was not established globally. For the global, SARS-CoV-2 and HIV-1 contrasts, the corrected 90% confidence intervals were too wide to fit within the equivalence bounds, irrespective of the point estimates. The three HCMV contrasts had sufficiently narrow intervals. Equivalence was established for UL55 and UL73 (TOST, *p* < 10^–30^), where both methods exceeded 0.997 macro-averaged F1. For US28, the difference favored the discriminative component by 0.042 F1 units and lay outside the equivalence bounds.

Considered jointly across all nine methods, the mean ranks over the 45 evaluation-unit by *m* blocks provide a descriptive ordering (Table 4). Chi-squared ranks first and the discriminative component second, a gap of 0.40 rank units.

Performance patterns varied between viruses, and the aggregate result conceals differences of opposite sign between datasets. For HCMV, all information-theoretic methods achieved near-perfect classification on UL55 and UL73 (F1 > 0.99), while on US28 they ranged from 0.926 to 0.969. The clear genotype-defining polymorphisms of UL55 and UL73 provide unambiguous discriminative signals readily captured by multiple approaches, and the discriminative component was statistically indistinguishable from chi-squared there, both methods operating at the ceiling of the task. On US28, the only unit where the two methods separate in favor of the discriminative component, the advantage was substantial (ΔF1 = + 0.042, 95% CI [+ 0.036, + 0.049], *p* < 0.001).

For HIV-1, characterized by extreme genetic diversity and complex subtype structure, the discriminative component achieved a mean F1 of 0.771, comparable to chi-squared (0.780) and NMI (0.771), and outperformed Cramér’s V (0.369) and ANOVA (0.623). Both paired differences were significant after Holm correction (adjusted *p* < 0.001 and *p* = 0.037 respectively), with full estimates reported in S5 Table. The difference with chi-squared was not statistically significant (ΔF1 =−0.009, 95% CI [−0.060,+0.043], *p* = 1.000). SARS-CoV-2 presented the most challenging discrimination task despite its lower diversity, due to the 19-class structure with highly imbalanced representation (113–101,757 sequences per variant). The discriminative component achieved mean F1 of 0.667, above MI (0.601), NMI (0.618), Cramér’s V (0.539) and ANOVA (0.353), but below chi-squared (0.676). None of these five differences is resolved after correction, including the one against chi-squared, where the corrected resampled test (ΔF1 =−0.009, 95% CI [−0.085,+0.067], *p* = 1.000) leaves the sign undetermined. For SARS-CoV-2, classification performance improved rapidly with increasing *m*: from F1 = 0.186 with a single position to F1 = 0.965 with 25 positions, which is consistent with discriminative variants being distributed across multiple genomic regions rather than concentrated at single sites.

Stratified by *m* and averaged over the five evaluation units, no selection size separates the discriminative component from chi-squared after correction (S5 Table). Within US28, significant differences occurred at *m* = 2 (+ 0.198 F1 units) and *m* = 3 (+ 0.202). No other unit showed a significant difference at any selection size (S5 Table). At *m* = 1, where redundancy between selected positions cannot contribute, the discriminative component and chi-squared reached nearly identical aggregate mean F1 values, 0.657 and 0.655.

Across the five evaluation units, point estimates against chi-squared ranged from −0.009 to + 0.042 F1 units. US28 was the only unit with a statistically significant difference, in favor of the discriminative component. The three negative point estimates were not statistically significant, and the aggregate value of + 0.005 reflects effects of opposite sign rather than a consistent advantage. The discriminative component is therefore not separated from chi-squared in aggregate, and no method dominates across the five evaluation units.

#### Selection agreement.

Because several methods reach comparable classification performance, we examined whether they do so by selecting the same positions (S7 Table). The methods did not produce identical top-*m* selections overall. Averaged across the five evaluation units and the nine values of *m*, mean Jaccard overlap with the discriminative component was 0.71 for MI and 0.69 for NMI, 0.59 for chi-squared and odds ratio, 0.54 for the normalized symmetric chi-squared divergence, and 0.36 and 0.32 for ANOVA and Cramér’s V.

Overlap with chi-squared was dataset-dependent: 0.79 across the three HCMV genes, where both methods approach the classification ceiling, against 0.29 for SARS-CoV-2 and HIV-1 (0.20 for HIV-1 alone). The complete score vectors separate two patterns. On HIV-1 the disagreement runs through the whole ranking, the Spearman correlation with chi-squared being 0.68. On SARS-CoV-2 the two criteria order the complete set of 3,027 positions almost identically, at 0.98, yet reach a mean top-*m* Jaccard index of only 0.37, sharing about half the positions each of them retains, so their disagreement is concentrated at the head of the ranking. Benchmark classification performance differed by 0.009 F1 units on SARS-CoV-2 and 0.009 on HIV-1, while the full-data top-*m* selections were not identical. Similar benchmark performance therefore should not be read as evidence that the criteria produce the same full-data selections.

#### Cross-dataset comparability.

Beyond classification performance, score scales differed across criteria. KSS discriminative values remained within the theoretical [0,1] bounds across all three viruses, with consistent empirical ranges over the benchmark universe used for method comparison: SARS-CoV-2 [0.00, 0.96], HIV-1 [0.01, 0.95], HCMV UL55 [0.00, 0.92], UL73 [0.00, 0.98], and US28 [0.00, 0.94] (Fig 2, right panels). For the three HCMV genes that universe also holds 81 reference-only windows at which no sequence varies. They score zero and are omitted from the integrated output, which is why those three lower bounds are exactly zero.

Maximum chi-squared values differed by more than three orders of magnitude between datasets (HCMV UL55: 1,197 vs. SARS-CoV-2: $1.73\times 10^{6}$). ANOVA exhibited even more extreme variation, with maximum values reaching 10^10^ across datasets. While theoretically bounded methods (NMI, Cramér’s V) showed more constrained ranges, their empirical distributions varied substantially across datasets. NMI ranged from [0, 0.65] for HIV-1 to [0, 1.0] for HCMV UL55, meaning that a score of 0.5 represents a near-maximum value in one context but a moderate value in another. The normalized symmetric chi-squared divergence and Cramér’s V both reached 1.00 on UL55 and UL73, whereas their maxima on SARS-CoV-2 were 0.87 and 0.89, respectively.

The bounded discriminative-component range avoids the scale drift seen for chi-squared or ANOVA, but it does not by itself establish that a given value carries the same meaning in two viruses. Position-level null thresholds differ between datasets, and the gap is carried by SARS-CoV-2: the 95th percentile of the conditional null has a median of 0.072 for HCMV and 0.065 for HIV-1, against 0.0055 for SARS-CoV-2, a gap of 0.067 between the extreme medians. The highest threshold at any of the 4,197 positions is 0.126 at the nominal margins and rises to 0.185, 0.273 and 0.455 as the margins are reduced, so a fixed cutoff cannot be treated as a dataset-independent calibration. The twelve largest discriminative-component scores in each dataset, which range from 0.834 to 0.975, exceeded their own position-specific thresholds at the nominal margins and at every reduction, clearing the highest threshold of any dataset by a factor of at least 1.8. A score of 0.7, below every reported top twelve value, would likewise exceed every estimated threshold across the three datasets, including under the reduced margins, which establishes separation from chance rather than equal discriminatory or biological meaning. The low end shows the lack of calibration most clearly: 0.05 exceeds the threshold of every SARS-CoV-2 position and of 6.5% of HCMV positions. The much lower threshold of SARS-CoV-2 is consistent with its much larger sample size, the null concentrating at lower scores as finite-sample fluctuations under independence shrink, although it depends on the whole set of margins and not on sample size alone. The bounded scale supports within-dataset ranking, but because the three classification schemes capture lineages, subtypes and genotypes at different evolutionary depths, it does not define a universal threshold or make score magnitudes statistically or biologically interchangeable across datasets.

#### Sensitivity to the prevalence threshold.

To test whether the prevalence threshold drives the final prioritization, we swept *t* from 0.10 to 0.50 on all three datasets, recompiling each dataset from its raw mutation records at every value. The set of scored positions is the same at every *t*, which makes the complete score vectors directly comparable between values. On HIV-1, the dataset the adjustment applies to, 0.25 and 0.33 give integrated KSS standard deviations of 0.1212 and 0.1211, share eleven of their twelve top-ranked positions, and correlate at a Spearman of 0.97 over the complete score vector. The two datasets that keep the default are close to inert, the twelve top SARS-CoV-2 positions being identical at every value of *t*. Score variances, selection overlaps and rank correlations across the whole range, for every dataset, are given in S4 Table. The adjusted *t* therefore moves at most one of twelve prioritized positions, on the single dataset it applies to.

### MIYATA_EVO matrix optimization

Genetic algorithm optimization of the MIYATA matrix stopped after 625 generations under the early-stopping rule, yielding MIYATA_EVO with composite value 4.578, a 28.4% increase over the original. MIYATA itself achieved the highest composite value among unmodified matrices, 3.566 (Table 5).

**Table 5:** MIYATA_EVO optimization and comparison with top-ranked matrices.

Rank	Matrix	Hydro.	Polar.	Volume	Area	pI	In-out	Aroma.	MinCodD	Composite
1	MIYATA_EVO	0.849	0.791	0.286	0.764	0.419	0.773	0.284	0.412	4.578
2	MIYATA	0.738	0.727	0.398	0.554	0.130	0.471	0.277	0.270	3.566
3	FENG	0.617	0.628	0.216	0.524	0.163	0.416	0.253	0.707	3.525
4	rtREV	0.709	0.682	0.280	0.622	0.055	0.501	0.287	0.312	3.449
5	BENNER74	0.697	0.666	0.302	0.621	0.006	0.433	0.289	0.427	3.441
6	BENNER22	0.674	0.638	0.283	0.550	0.031	0.397	0.321	0.544	3.439
7	GONNET1992	0.705	0.670	0.295	0.629	-0.001	0.440	0.276	0.388	3.402
8	VTML250	0.677	0.638	0.311	0.595	0.021	0.438	0.302	0.370	3.351
9	VTML200	0.664	0.630	0.323	0.575	0.019	0.416	0.309	0.383	3.319
10	JONES	0.626	0.600	0.245	0.503	0.043	0.389	0.303	0.601	3.309

**Rank:** Overall rank including MIYATA_EVO, the product of the optimization; S1 Table reports ranks among candidate matrices only. **Hydro.:** Hydrophobicity. **Polar.:** Polarity. **Area:** Fraction of accessible surface area lost upon folding. **pI:** Isoelectric point. **In-out:** Interior-exterior transfer propensity. **Aroma.:** Aromaphilicity. **MinCodD:** Minimum codon distance. **Composite:** Sum of all eight correlation coefficients. All values represent Spearman rank correlation coefficients (ρ) calculated across 190 amino acid pairs. MIYATA_EVO was generated via genetic algorithm optimization (population = 1,000, max generations = 10,000, mutation rate = 0.1, crossover probability = 0.5) operating on 190 unique pairs (upper triangle) to maximize composite score while enforcing symmetry by construction. Early stopping after 625 generations following 100 consecutive generations without improvement. Increase over MIYATA: + 28.4%.

MIYATA_EVO increased seven of the eight correlations relative to the original MIYATA matrix (Table 5). The largest relative gains occurred for isoelectric point (+221.8%), interior-exterior transfer propensity (+64.1%), MinCodD (+52.6%), accessible surface area (+37.9%), and hydrophobicity (+15.0%). Polarity (+8.8%) and aromaphilicity (+2.2%) showed smaller improvements. Side chain volume decreased (ρ=0.286 vs. 0.398, −28.1%). Isoelectric point, where several of the top-ranked matrices show essentially no correlation, is the characteristic the optimization improved most in relative terms. That increase measures how much more closely the optimized matrix aligns with the predefined mutational-impact construct.

Among the unmodified candidates, the physicochemical matrices MIYATA and FENG [63] hold the two highest composite values, followed by the virus-derived rtREV model and the evolutionary BENNER74 and BENNER22 matrices (Table 5). The leading candidates therefore span both physicochemical and replacement-rate families.

Across the four conversion divergences examined, five of the six rate models remained within two ranks of their positions at δ=2.5, whereas WAG varied most, from 10th to 17th overall (S1 Table). The overall fourth- and fifth-placed matrices differed by less than 0.01 in composite value, so their relative ordering is descriptive.

Frequency-based matrices such as PAM and BLOSUM summarize replacement patterns observed in protein sequence data [26,27], whereas MIYATA_EVO provides the mutational component with distances optimized against seven physicochemical properties and the minimum codon distance. The composite ranking therefore assesses suitability for that role rather than evolutionary model adequacy.

### Protein-component calibration against UniProt annotation scores

Across 17,471 viral proteins, the protein characterization score $S_{p}$ ranged from 0.000 to 1.000 (mean = 0.313, SD = 0.217, median = 0.300). Proteins were distributed across UniProt’s five annotation scores [64]: score 1 (25.1%), score 2 (28.3%), score 3 (23.6%), score 4 (12.2%), and score 5 (10.8%).

Table 6 summarizes the relationship between $S_{p}$ and the UniProt annotation score. Because the two quantities are not independent, their association is treated as a descriptive calibration rather than independent validation. The comparison assesses whether a continuous score built from ten weighted categories orders proteins as the five-level UniProt measure does, and how much resolution it adds within each category. Spearman rank correlation was ρ=0.900 (95% CI: 0.897-0.903) and Kendall’s tau was τ=0.775 (95% CI: 0.765-0.785), the latter accounting for tied ranks. Mean $S_{p}$ increases monotonically across scores, from 0.066 in score 1 to 0.668 in score 5 (Table 6).

**Table 6:** Protein-component calibration against UniProt annotation scores.

UniProt annotation score	*n*	Mean	SD	Range	CV (%)
1	4,387	0.066	0.063	0.000-0.420	94.44
2	4,938	0.236	0.092	0.000-0.625	39.08
3	4,121	0.409	0.109	0.050-0.800	26.69
4	2,140	0.503	0.104	0.175-0.945	20.79
5	1,885	0.668	0.147	0.075-1.000	22.03
**Overall**: 17,471 proteins, mean = 0.313 ± 0.217, median = 0.300, range = 0.000-1.000					

Distribution of $S_{p}$ across 17,471 viral proteins stratified by UniProt annotation score. Because the two measures draw on overlapping UniProt annotation content, the comparison is descriptive rather than an independent validation. ***n*:** Number of proteins. **Mean:** Average $S_{p}$. **SD:** Standard deviation. **Range:** Minimum–maximum $S_{p}$. **CV:** Coefficient of variation. UniProt annotation scores range from 1 (basic annotation) to 5 (best-annotated entries) [64]. Correlation statistics: Spearman’s ρ=0.900 (95% CI: 0.897-0.903); Kendall’s τ=0.775 (95% CI: 0.765-0.785).

Dispersion within each category is where the added resolution shows. Relative dispersion, measured by the coefficient of variation, is largest in score 1 (CV 94.44%), largely because its mean is close to zero. That category groups proteins to which the scoring rubric assigns $S_{p}=0.000$ with proteins carrying experimental evidence and a determined structure (up to $S_{p}=0.420$), a distinction the five-level scale cannot express. Relative dispersion narrows to a minimum at score 4 (20.79%). Table 6 reports the full distribution.

Using the strict threshold |Δ|>0.30 (where $\Delta =S_{p}$ minus the normalized UniProt annotation score), 18 proteins (0.1%) showed positive discordance, with $S_{p}$ above the normalized UniProt score, and 1,703 (9.7%) showed negative discordance. Both scores take discrete values, which places many proteins exactly on the threshold: a further 338 with |Δ|=0.30 are excluded from both sides. Under the chosen normalization, $S_{p}$ is on average 0.075 below the normalized UniProt annotation score, which partly accounts for this directional imbalance. UniProt applies qualitative curation standards emphasizing completeness and evidence quality [65], while the component scores the same records quantitatively, weighting functional annotations and experimental evidence most heavily.

Every positive-discordance protein (high $S_{p}$, low UniProt score) has experimental evidence at protein level (PE = 1) and a UniProt annotation score of 1 or 2, and 12 of the 18 (66.7%) have an experimentally determined 3D structure (S8 Table). Among the 20 most negative cases (S9 Table), one (5%) has an experimental structure and 14 (70%) are inferred by homology (PE = 3). GO coverage runs the other way within these selected extremes: the positive cases carry a median of two GO terms across the three ontologies, against nine among the most negative ones. For instance, the most extreme case of positive discordance is HCMV protein UL78 (P16751, Δ=+0.42), which possesses experimental evidence, seven structural/functional features, characterized post-translational modifications, and a cryo-EM structure [66] despite minimal UniProt annotation, with recent work demonstrating its G protein-coupled receptor activity [67]. Conversely, the most extreme case of negative discordance is Sulfolobus islandicus rod-shaped virus 1 protein 1070 (Q8QL26, Δ=−0.925), which received the maximum UniProt score yet a minimal $S_{p}$ due to predicted status (PE = 4), absent structure, and no Gene Ontology terms. These deliberately selected extremes illustrate how the two scoring frameworks weight protein-existence evidence, structural information and annotation completeness differently.

### Functional and discriminative site validation

#### Concordance with published sites.

Top-ranked KSS positions showed concordance with literature-documented functional or strain-distinguishing loci across all three viruses (Fig 3).

**Fig 3:**
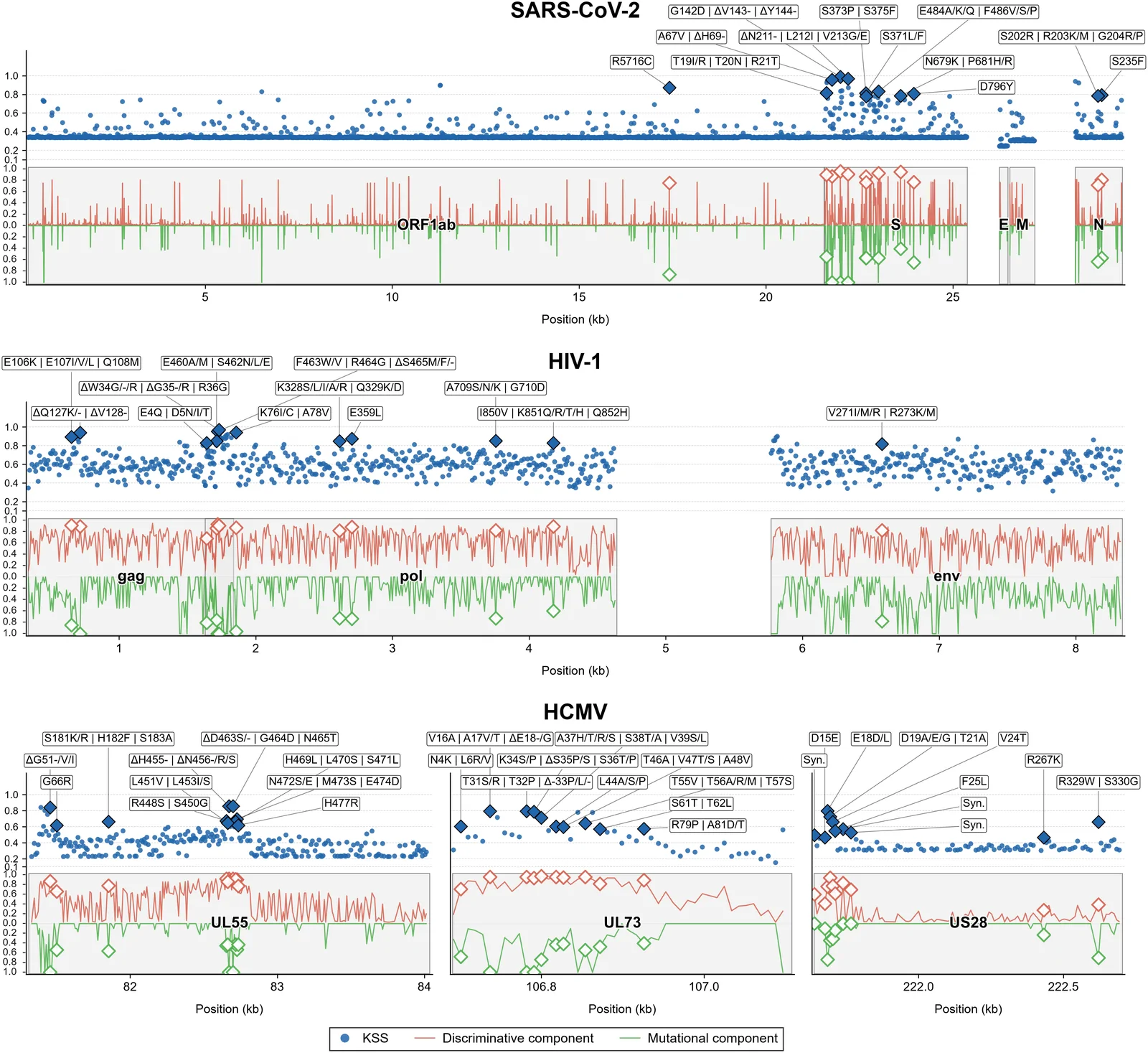
Functional and discriminative site validation across viruses. KSS (blue), discriminative component (red), and mutational component (green) across genomic coordinates for SARS-CoV-2, HIV-1, and HCMV. Gene annotations shown as gray boxes. Top-ranking sites are marked with diamond symbols on each score component. Top twelve positions for SARS-CoV-2 and HIV-1 (genome-wide) and top ten per gene for HCMV, selected with a maximum of three indel positions per selection. Labels give the amino acid changes a position carries; Δ marks an insertion or deletion, and Syn. denotes a position containing only synonymous substitutions. See S10–S12 Tables for detailed mutation listings.

For SARS-CoV-2, the twelve positions drawn in Fig 3, selected under the three-indel rule stated in that legend, span three genes with KSS from 0.78 to 0.99: nine in Spike (S), two in Nucleocapsid (N), and one in ORF1ab. The three-indel rule changes what is drawn: only four of these twelve are among the twelve highest-scoring positions of the unfiltered ranking, where eleven of the twelve are insertions or deletions. The three highest-scoring positions, all involving indels (mutational score = 1.0), localized to the Spike N-terminal domain (NTD). Positions 142–144 (KSS = 0.99; G142D, V143del, Y144del) and 211–213 (KSS = 0.97; N211del, L212I, V213G) lie within the NTD antigenic supersite targeted by neutralizing antibodies [68–70]. The changes at the first are lineage-associated across Alpha, Delta and Omicron, whereas the second separates Omicron sublineages. Positions 67–69 (KSS = 0.96; H69del, A67V) correspond to recurrent deletion region 1 (RDR1), associated with immune escape and antibody evasion [68,71].

The nine substitution-based positions extended across six functionally distinct genomic regions. Three positions clustered within the receptor-binding domain (RBD). The E484A/F486V position (KSS = 0.83) includes E484, an immune escape site associated with reduced neutralization by convalescent and vaccine sera [69]. At position 371 (KSS = 0.81), 99.8% of S371L-bearing sequences belonged to BA.1, whereas S371F-bearing sequences were distributed mainly among BA.2 and later Omicron lineages (S10 Table). Both substitutions broadly reduce neutralization by receptor-binding-domain-directed monoclonal antibodies [72,73]. S373P/S375F (KSS = 0.78) completes the 371–375 mutation cluster characteristic of Omicron [71]. At the furin cleavage site, P681H/R (KSS = 0.79) enhances Spike processing and defines Alpha (P681H), Delta (P681R), and subsequent lineages [71]. In the NTD, T19I (KSS = 0.82) occurred in *k*-mer variants whose carriers were concentrated across BA.2 and several later Omicron lineages. It is among the Spike alterations distinguishing BA.2 from BA.1 and was reported to subtly impair neutralization by class 1 RBD antibodies [73]. In the S2 subunit, D796Y-bearing sequences (KSS = 0.81) were distributed across several Omicron lineages. The substitution lies immediately upstream of the fusion peptide and markedly reduces binding of infection- and vaccination-induced antibodies to linear Spike peptides spanning D796 [74]. In the Nucleocapsid, R203K/G204R (KSS = 0.79) in the serine/arginine-rich linker represents variant-defining mutations associated with increased viral fitness [75], while S235F-bearing sequences (KSS = 0.79) were almost exclusively B.1.1.7 (S10 Table). That substitution eliminated IgG binding to the corresponding linear nucleocapsid epitope in a peptide-array assay [76]. The ORF1ab position R5716C (KSS = 0.87), corresponding to NSP13 R392C in the helicase domain, distinguishes all post-BA.1 Omicron sublineages [77].

For HIV-1, the twelve top-ranking positions (KSS = 0.82–0.97) distributed across all three genes: seven in pol, four in gag, and one in env. The selection comprised three indels and nine substitutions, reflecting the indel constraint applied to a genome with extensive subtype-specific length polymorphisms.

In pol, KSS identified positions spanning four functional regions of the Gag-Pol polyprotein: transframe peptide (TFP), protease, reverse transcriptase (RT), and integrase. The highest-scoring pol position (KSS = 0.97; W34del, W34R, R36G) localizes to the transframe peptide (TFP/p6*), a region involved in Gag-Pol polyprotein processing and protease autoactivation [78], where subtype-specific deletions and substitutions distinguish multiple subtypes. Position 5 (KSS = 0.83; D5N, D5I) also maps to the TFP, in a region exhibiting subtype-specific variation [78]. In the protease, position 76 in pol (corresponding to protease standard position 20; KSS = 0.94; K76I) maps to a site where K20R/M/N are documented as PI-selected mutations [79]. In RT, K328 (KSS = 0.85; corresponding to RT position 173) maps to the palm subdomain [80], as does the window anchored at position 358 (KSS = 0.87; RT position 203), at a residue where E203D/K substitutions are associated with nucleoside analogue exposure [81,82]. In this dataset the discriminative signal at that position arose predominantly from synonymous subtype-distinguishing variation. The only non-synonymous change in the window, E359L (RT E204L), occurred exclusively in group O. A709S/N/K (KSS = 0.85; RT position 554) maps to the RNase H domain, within a region implicated in nucleoside analogue resistance [80,83]. In the integrase domain, K851Q (KSS = 0.83; integrase position 136) localizes to the catalytic core domain near the DDE active site motif (D64, D116, E152) [84]. Position K136 has been experimentally implicated in viral DNA binding [85], and the K136Q polymorphism is associated with prior antiretroviral drug exposure [86], as cataloged in the HIV Mutation Browser [87].

In gag, KSS identified positions in two functionally distinct domains. In the matrix (MA/p17), E107I (KSS = 0.89) maps to helix 5, which also contains E99, a residue required for Env incorporation into virions [88]. Position Q127del/V128del (KSS = 0.94) falls within an HLA-restricted cytotoxic T-lymphocyte (CTL) epitope (positions 124–132) where Q127 exhibits subtype-specific polymorphism reflecting population-level HLA-driven immune selection [89]. In the p6 domain, S465del (KSS = 0.95), the highest gag position, and E460A (KSS = 0.85) map to the region immediately downstream of the PTAP late domain, which recruits Tsg101 for budding through the endosomal sorting complex required for transport (ESCRT) [90], and next to the FRFG motif essential for Vpr incorporation into virions [91]. The p6 domain exhibits subtype-specific insertions and deletions, with PTAP motif duplications in subtype C conferring replication advantages [92,93].

In env, the single top-ranking position V271I/M/R (KSS = 0.82) maps to the gp120 C2 conserved region [94], where the amino acid variant shows strong subtype association: V271I predominates in CRF01_AE, C, D, and multiple BC recombinants, V271M is carried mainly by A1, B and A6, while V271R is exclusive to group O (S11 Table).

For HCMV, the ten highest-ranking positions in each gene were compared with published genotype-defining polymorphisms for UL55 and UL73 and documented functional regions in US28. In UL55, the two highest-ranking positions (KSS = 0.86; N456del, H455del, D463del) localized to the hypervariable region flanking the furin cleavage site at codon 461, where deletions match genotype-defining criteria documented by Chou and Dennison [48]: group 2 carries N456del, while group 3 carries H455del and D463del. Additional top-ranking positions at codons 51 and 66 identified discriminative N-terminal loci outside the classical gB genotyping scheme [48]. In UL73, top positions (KSS = 0.57–0.79) mapped predominantly to the N-terminal domain (codons 4–79), matching genotyping criteria established by Pignatelli et al. [49]. Key polymorphisms included genotype-defining deletions at E18 and S33, with E18del characterizing genotype 4 subgroups and S33del distinguishing genotype 2, within a region clinically associated with symptomatic congenital infection [95].

In US28, the top positions (KSS = 0.47–0.80) distributed across functionally distinct domains: N-terminal extracellular positions (E18L, D19A) map to the chemokine-binding domain implicated in immune evasion [47,96], while C-terminal intracellular position R329W (KSS = 0.66) maps to the signaling-relevant C-terminal tail, a region implicated in constitutive G-protein coupling [97]. US28 contained exclusively substitution-based top positions (0% indels), contrasting with UL55 (30% indels) and UL73 (30% indels). Three positions (KSS = 0.50–0.57) contained only synonymous substitutions and had a mutational score of zero. Because $S_{p}$ is constant within US28, the differences among these positions came entirely from the discriminative component.

In this targeted literature cross-reference, all 12 reported SARS-CoV-2 positions mapped to cited immune escape, antibody evasion or variant-defining sites, and all 12 HIV-1 positions mapped to documented drug resistance, structural, or immune selection regions. The HCMV analysis recovered established UL55 and UL73 genotype-defining polymorphisms together with documented US28 regions (S10–S12 Tables). The cited site-specific annotations were not used to construct the position scores. The lineage and subtype distributions reported above and in those tables are descriptive summaries of the same class labels used by the discriminative component and therefore do not constitute independent corroboration. The three HCMV comparisons are bounded more tightly still: the locus-specific genotype labels of UL55, UL73 and US28 were themselves defined from the polymorphism schemes of Chou and Dennison [48], Pignatelli [49] and Arav-Boger [47], the same works the recovered positions are compared with, so that recovery is a consistency check on label-defining signal rather than external support. Among the HCMV references, only those describing US28 function [96,97] lie outside the definition of its genotypes. These HCMV datasets were larger than those of the original studies: 443 US28 sequences versus 55 in Arav-Boger [47] (8.1-fold increase), 399 UL55 sequences versus 14 in Chou and Dennison [48] (28.5-fold increase), and 646 UL73 sequences versus 42 in Pignatelli [49] (15.4-fold increase). The corresponding genotype-defining polymorphisms remained among the highest-ranked positions in these expanded datasets.

#### Reference-specific error rates against external annotation.

The annotation layers evaluated here do not form an exhaustive catalog of functionally relevant viral positions, so the rates below are reference-specific rather than absolute. Each is reported with its evaluable universe and chance expectation. The genome-wide layers hold between 645 and 2,769 positives, so at *m* = 12 the false negative rate exceeds 98% regardless of the ranking method: a selection of twelve can recover at most twelve of them.

Common-mutation membership is saturated. Of the 3,027 *k*-mers scored across the five SARS-CoV-2 genes, 2,769 are positives, a prevalence of 91.5%, so all twelve highest-scoring *k*-mers are positives and precision at *m* = 12 is 1.00 with a false discovery rate of 0. The result is uninformative: the enrichment is 1.09-fold and the probability of drawing at least as many positives by chance is *p* = 0.343. At *m* = 100 all hundred are positives as well and the enrichment is unchanged at 1.09-fold, though the draw is no longer likely by chance ($p=1.2\times 10^{-4}$): the selection size moves the probability, not the enrichment. This reflects the construction of the reference: COV2Var admits a mutation if it exceeds 1% frequency in at least one Pango lineage, occurs at least twice within that lineage, and is present in at least two lineages. Of the 9,832 cataloged mutations, 40.7% are synonymous, and the admission rule is prevalence-based, like the KSS filter, rather than site-specific functional evidence.

The five selection-based layers comprise the MEME, FEL and FUBAR calls separately, their union, called by at least one test, and their intersection, called by all three, and are evaluated on the 2,031 *k*-mers represented in the distributed files. The union and the intersection are a broad and a restrictive reading of the same call set, not two further tests. COV2Var provides only the significant MEME and FEL calls, whereas its FUBAR file also lists non-significant sites. Across these five layers, prevalence ranges from 31.8% to 81.5% in that universe. The largest enrichments at every selection size are those of FEL and the intersection of the three tests: against the intersection, which flags 645 of those *k*-mers, the hundred highest-scoring contain 77 positives, and against FEL, which flags 706, they contain 79. On the gene-matched null the two layers expect 42.2 and 44.5 positives, against 31.8 and 34.8 if the selection were drawn from the universe at random. The observed counts exceeded both the flat and gene-matched expectations. Under the flat null, which treats evaluable *k*-mers as exchangeable, the corresponding nominal hypergeometric probabilities were $p\leq 1.9\times 10^{-20}$ at *m* = 100. Because neighboring *k*-mers and reference calls may be spatially dependent, these probabilities are not interpreted as calibrated evidence at their numerical precision. The observed-to-expected counts and enrichment estimates are therefore the primary summaries. The other three layers have higher prevalences, 63.7%, 66.2% and 81.5%, and smaller enrichments across selection sizes, 1.21 to 1.57-fold against 2.27 to 3.15-fold for these two. These rates remain conditioned on variation, a position under selection being necessarily variable, as are the positions this component prioritizes. The three procedures are not independent sources of evidence either: they analyze the same alignment, and MEME tests branch-specific episodic selection where FEL and FUBAR test pervasive site-specific selection. Their call sets are therefore related reference layers rather than independent corroboration.

The experimental layers are local: both deep mutational scanning layers assayed 68 residues of the receptor-binding domain, leaving 45 evaluable *k*-mers for antibody escape, which is saturated at 93.3% prevalence, and 44 for ACE2 binding, where nine of the ten highest-scoring evaluable *k*-mers are positives against 5.0 expected by chance and the ranking reaches AUROC 0.855 ($p=2.8\times 10^{-5}$). Site-level fitness estimates [98] were examined as an exploratory characterization of evolutionary constraint and were not used as a validation reference for the integrated KSS (S6 Table). Every layer, at every selection size its universe admits, with that universe and its chance expectation, is given in S6 Table.

Top-ranked *k*-mers are enriched for positives in several selection-based layers and in the ACE2-binding layer, but taken together these resources do not constitute a complete, genome-wide and unsaturated benchmark for the quantity this framework ranks.

### Computational cost

For a shared contingency-table procedure the per-position cost is *O*(*N* + *VK*), for the discriminative component, chi-squared and mutual information alike. The reference implementation does not attain that bound: it deduplicates the *k*-mer configurations with a row sort and passes over the class labels once per distinct *k*-mer and once per class, which costs O(NVlogN+N(V+K)). Measured across the eleven genes, the discriminative score takes about 0.33 ms per position at 443 sequences (HCMV US28), 12.7 to 15.0 ms at 12,223 (the three HIV-1 genes) and 0.27 to 0.30 s at 278,738 (the five SARS-CoV-2 genes). Across sequence counts from 399 to 278,738, time normalized by *N*(*V* + *K*) varied only 2.4-fold, broadly consistent with near-linear scaling in *N*(*V* + *K*), although these measurements do not isolate the logarithmic term from implementation constants. The discriminative score ranged from 0.54 to 3.4 times the execution time of chi-squared and was comparable to mutual information. That range compares implementations rather than criteria, since chi-squared and mutual information use optimized library routines.

The mutational component stays under 6 microseconds per position, and the protein characterization score is a per-gene constant computed once from UniProt. Timing both stages of a complete run shows where the cost sits: on SARS-CoV-2 E, preprocessing takes 48 s against 19 s for the whole scoring layer, 72% of the two stages together, and on HCMV US28 0.48 s against 0.06 s, 88%. The implementation can distribute the independent sequence alignments across multiple worker processes, which shortens that shared stage without changing the order of any step. Because the translation, alignment and *k*-mer extraction that build the contingency tables are shared by any method operating on the same position-indexed matrix, chi-squared included, the mutational and protein information adds little marginal local computation beyond a bare discriminative filter.

### Limitations and future directions

The framework operates on coding sequences only, since translation, protein-level alignment and the codon mapping that connects nucleotide *k*-mers to amino acid changes all presuppose a coding sequence. Regions without an associated protein, among them untranslated regions and viral non-coding RNAs, can carry regulatory functions, but they are not assigned a KSS. Viral genomes are nevertheless coding-dense overall: in a survey of complete genomes, median non-coding fractions were about 10% for DNA viruses and 6% for RNA viruses, with substantial variation among viral groups [99]. Regulatory elements embedded within coding sequences remain visible to the discriminative component through their nucleotide variation, including synonymous changes, but their RNA-level function is not modeled. Extending the framework beyond coding sequences would require corresponding RNA-level descriptors and remains future work.

The number of genes analyzed also shapes what the protein component can contribute. The effect of adding or removing genes was not tested, so the gene sets used here should not be read as optimal. For a new virus the criteria are set by the study rather than by KSS: any coding gene that falls within the classification scope and carries enough class-labeled sequences to estimate variant prevalence reliably at the chosen *t* can be included, without preselecting genes for sites already known to distinguish classes. The protein component is constrained differently: because $S_{p}$ is constant within a gene, it can affect only selections pooled across genes. Its coverage is that of UniProt: because annotation is shaped by uneven research attention [100–102], the component favors well-characterized proteins and may underweight poorly studied ones, which is inherent to any annotation-dependent framework and implies that such analyses should be re-evaluated as the databases are updated. Setting $w_{p}=0$ removes the component and the UniProt lookup with it, thereby measuring how much the annotation prior contributes to the reported selections. At this setting the reported SARS-CoV-2 selection remained unchanged, its three contributing genes having the maximum characterization score. The three HCMV selections are unaffected by construction, their genes being ranked separately. For HIV-1, three of twelve positions changed in the unfiltered ranking and two in the selection shown in Fig 3. Because the three components are also reported separately, positions with high $S_{d}$ and $S_{m}$ but low $S_{p}$ can flag discriminating variation with assessed biophysical impact in less-characterized proteins, making them candidates for follow-up rather than positions promoted by the default annotation-weighted ranking.

Parameterization was only partly explored: *t* was swept and $w_{p}=0$ was ablated, but the *k*-mer length, $w_{d}$, $w_{m}$ and the protein scoring category weights (S2 Table) were set from domain knowledge rather than formally optimized. The mutational component provides biophysical granularity for amino acid substitutions, but assigns a uniform configurable score to insertions and deletions (default 1.0) regardless of structural context, so it cannot distinguish small in-frame indels in flexible loop regions from functionally critical deletions in antigenic domains. Context-dependent scoring, informed by protein structural data or disorder predictions, would address this. Results also depend on the choice of reference genome, as positional coordinates and mutation calls are defined relative to a single reference per virus. Alternative references could shift individual position rankings. Automated CDS extraction from whole-genome inputs and codon-aware alignment for divergent sequences remain implementation extensions.

This assessment covers three viruses and five evaluation units. Resampling refines estimates within those systems but does not establish generalization to untested viral architectures such as negative-sense RNA viruses or segmented genomes. Functional validation rests on retrospective concordance with published literature, a common strategy for a prioritization framework, rather than on prospective prediction, so it establishes agreement within the references consulted rather than predictive value. High-scoring positions without current literature support may represent genuinely relevant sites not yet characterized, or class-associated variation with no known functional role, and only prospective experimental validation would distinguish the two.

The discriminative component scores each position independently, so a position’s score does not depend on which others are selected. That makes it usable as a feature-ranking step to construct compact panels for classification among predefined viral classes. In our benchmark, the 25 highest-ranked positions, the largest panel evaluated, reached a mean macro-averaged F1 of 0.989 across the five evaluation units, with unit means from 0.965 for SARS-CoV-2 to 1.000 for HCMV US28. That mean rose from 0.972 at *m* = 20, driven by SARS-CoV-2 at 0.884 there, so the experiment established neither a performance plateau nor an optimal panel size. The same independence means such a panel is not optimized jointly: epistatic and combinatorial interactions between positions are not captured, and two non-overlapping windows can carry nearly the same class signal. Replacing the independent top-*m* ranking with a set-level objective that penalizes redundant signal could identify a smaller panel. Its size would be chosen under nested validation and assessed on independent sequences, and each retained position remains traceable to a named genomic coordinate.

## Conclusion

This study presents KSS, a framework integrating discriminative power with biological context for viral *k*-mer prioritization. Across SARS-CoV-2, HIV-1, and HCMV, KSS prioritized genomic positions that matched established functional or strain-distinguishing loci, while providing interpretable values in the [0,1] interval for ranking positions within a defined classification task.

The three-component architecture addresses limitations of existing *k*-mer selection methods by adding biological context to discriminative ranking. The discriminative component reached a mean F1 of 0.881 against 0.876 for chi-squared, with no statistically significant global difference, and significantly outperformed three of the seven established baselines. Its bounded [0,1] scale supports integration with the mutational and protein components. The MIYATA_EVO matrix, optimized using a genetic algorithm, increased the composite agreement score across eight amino acid characteristics by 28.4% over the original MIYATA matrix. The protein characterization score tracked the UniProt annotation score closely (ρ=0.900, τ=0.775) and provided finer numerical resolution among proteins within the same five-level category. Because both read the same annotations, that comparison is a calibration rather than an independent validation. Literature cross-referencing found that the reported top-ranked positions corresponded to established variant-defining or functionally characterized loci across the three viruses (12/12 for SARS-CoV-2, 12/12 for HIV-1, and recovery of established UL55 and UL73 genotype-defining polymorphisms for HCMV). The cited site-specific annotations were not used to construct the position scores, although the HCMV genotype labels were defined from the polymorphisms recovered there, which makes that recovery a consistency check rather than external support.

The modular design and interpretable score decomposition support applications ranging from routine strain classification to the prioritization of mutations for follow-up characterization. In a complete run, the analysis of over 290,000 sequences across 11 genes finished in under 24 hours using a single worker on the machine described in Materials and methods, without the parallelization the alignment stage allows. Runs of this size are therefore compatible with large-scale molecular surveillance.

## Supporting information

S1 TableSubstitution matrices evaluated for the mutational component.Sheet 1: the 37 candidate matrices tested, ordered by composite score and numbered by their rank among candidates, with the Spearman correlation obtained for each of the eight amino acid characteristics, the family each matrix belongs to (evolutionary substitution or replacement rate, physicochemical distance, alignment scoring, replacement rate estimated from viral alignments, or derived from the genetic code), the source its values were matched to, and the source used to verify it where one applies. For the seven matrices added in revision the file actually read is known and pinned. For the thirty of the base panel no acquisition record survives, so the column reports where the same values are found rather than a route: thirteen match Biopython alone; four match AAindex entries absent from the other sources, namely BLOSUM62 [27], GRANTHAM [36], MIYATA [18] and PAM120 [103]; two match an AAindex entry also present in the Spaln tables; ten are identical in Biopython and AAindex and cannot be told apart on values; and SNEATH matches the dissimilarity table published by Sneath [104]. Ranks in this sheet exclude MIYATA_EVO and therefore sit one position below the overall ranks quoted in Table 5, which includes it. Sheet 2: the same quantities for MIYATA_EVO, which is the product of the genetic algorithm rather than a candidate matrix and is therefore reported separately. Sheet 3: the six rate models at each of the four conversion divergences δ, with the composite, the overall rank, the rank among candidates, the number of distinct off-diagonal values the integer rounding leaves, and the rank correlation against the reference divergence.(XLSX)

S2 Table Components and weights of the protein characterization score. Ten annotation categories with differential weights reflecting their relative contribution to protein characterization. Categories include Gene Ontology terms (molecular function, biological processes, cellular component), protein existence evidence, structural features, protein-protein interactions, post-translational modifications, 3D structures, drug target information, and literature references. The last column names the UniProt field or the cross-referenced resource each category is read from; all are taken from the protein’s UniProt entry rather than from the referenced databases directly. Total maximum: 100 points.
(XLSX)

S3 Table Inventory of the classes used in each dataset. Sheet 1: for each dataset and each classification scope, the nomenclature the classes derive from, the number of classes and the total number of sequences. Sheet 2: every class individually, with its label, its number of sequences, the nomenclature it belongs to, and the nature of the class, which distinguishes Pango lineages from recombinant lineages and from the single label grouping two sister lineages for SARS-CoV-2, group M subtypes from circulating recombinant forms and from group O for HIV-1, and identifies the HCMV entries as locus-specific genotypes.
(XLSX)

S4 Table Sensitivity of the prioritization to the prevalence threshold *t.* For each dataset and each threshold from 0.10 to 0.50, the number of scored positions, the number of retained *k*-mers and how many of those are alternatives to the reference, the mean, standard deviation and variance of the KSS distribution, and two measures of stability against the threshold used in the manuscript (0.25 for SARS-CoV-2 and HCMV, 0.33 for HIV-1): the overlap of the selected positions, reported for the selection each dataset publishes, twelve pooled across genes for SARS-CoV-2 and HIV-1 and ten per gene for HCMV, and for a pooled top 12 and top 25 in every case, and the Spearman rank correlation of the complete score vector.
(XLSX)

S5 TablePaired statistical comparison of the discriminative component against baseline feature selection methods.Sheet 1: global contrasts of the discriminative component against each of the eight baselines, averaged over the five evaluation units within each iteration. Columns: mean paired difference in macro-F1, corrected and uncorrected standard errors, 95% confidence interval, corrected resampled *t*-test *p*-value [58] before and after Holm-Bonferroni adjustment, uncorrected Wilcoxon signed-rank *p*-value reported for comparison, Cohen’s $d_{z}$, win/loss counts over the 100 iterations, and TOST equivalence outcome at margins of 0.01 and 0.02 F1 units. Sheet 2: the same contrasts computed separately for each evaluation unit. Sheet 3: the discriminative component versus chi-squared stratified by the number of selected positions *m*, first averaged over the five evaluation units and then within each of them, since averaging conceals a difference confined to one unit. Sheet 4: descriptive mean-rank summary of the nine methods over the 45 blocks. Sheet 5: standard error of the global difference between the discriminative component and chi-squared as a function of the number of iterations used, contrasting the uncorrected estimate with the corrected estimate. The confidence intervals are pointwise intervals based on the corrected resampled standard error and are not adjusted for multiplicity, while Holm-Bonferroni adjustment applies to the reported corrected resampled *t*-test *p*-values.(XLSX)

S6 Table COV2Var reference-layer matrix for SARS-CoV-2 prioritization. Sheet 1: for each reference layer and each reported selection size, the evaluable universe and prevalence, the confusion counts, precision, false discovery rate, false positive rate, recall and false negative rate at *m*, the positives expected at *m* under a random draw and under a draw matching the gene composition of the selection, enrichment over prevalence, the hypergeometric probability of drawing at least as many positives and of drawing *m* positives out of *m*, both nominal under a null that treats evaluable *k*-mers as exchangeable, the longest prefix of the ranking containing no false positive, and the AUROC over the full ranking with its *p*-value. Layers are common-mutation membership; the MEME, FEL and FUBAR tests for diversifying selection taken singly, in union and in intersection, each restricted to the *k*-mers the selection analysis reports on; the two receptor-binding domain deep mutational scanning measurements, each restricted to the *k*-mers carrying a measured residue; the three effect-prediction layers, labeled as predictions and each restricted to the substitutions it scored; and the union of the admitted layers, with and without the membership layer. Sheet 2: which layers were admitted, restricted, reported without being admitted, or found inapplicable, and why, including the three produced by effect-prediction software, the descriptor layer excluded with them, and the one category the bulk download does not distribute.
(XLSX)

S7 Table Overlap between the positions selected by the discriminative component and by the baseline methods. Sheet 1: Jaccard index between the top-*m* positions retained by the discriminative component and by each of the seven deterministic baselines, averaged over the five evaluation units, for each value of *m* used in the classification protocol. The random draw is excluded, an overlap with it measuring nothing about agreement between criteria. Sheet 2: the same indices reported separately for each evaluation unit, with the number of shared positions. Sheet 3: Spearman correlations between the complete score vectors of the discriminative component and of each baseline, per evaluation unit. The vectors span the benchmark universe, which for the three HCMV genes holds 81 reference-only windows that score zero and are omitted from the integrated output, so the position counts of that sheet exceed the 385 HCMV positions the integrated output emits. The SARS-CoV-2 and HIV-1 universes carry no such window and are identical under both readings.
(XLSX)

S8 TableThe 18 positive discordance proteins (high $S_{p}$, low UniProt annotation score).Every protein with Δ>0.30. Columns: UniProt accession, annotation score (raw and normalized), $S_{p}$, discordance (Δ), protein name, organism, protein existence level, number of references, Gene Ontology annotations (MF, BP, CC), 3D structure availability, post-translational modifications, known interactions, and number of structural/functional features.(CSV)

S9 TableTop 20 negative discordance proteins (high UniProt annotation score, low $S_{p}$).The 20 most negative cases among the 1,703 proteins with Δ<−0.30. Same column structure as S8 Table.(CSV)

S10 Table SARS-CoV-2 top-ranking positions: detailed mutations and indel statistics. Top 12 genome-wide KSS positions under the three-indel selection rule (Sheet 1) expanded to one row per variant *k*-mer. Columns: rank, gene, gene position, KSS and component scores (discriminative, mutational, protein), reference *k*-mer, variant *k*-mer, amino acid changes with mutational impact scores, indel status, and variant/lineage distribution. The variant-distribution column reports, for each *k*-mer variant, the percentage of its carriers assigned to each class (variant, subtype, or genotype). Classes contributing less than 1% of carriers are omitted individually and summarized as “+ *n* others”. Sheet 2 provides per-gene indel statistics (ORF1ab, S, M, N, E): total positions analyzed, positions with indels, and indel percentage.
(XLSX)

S11 Table HIV-1 top-ranking positions: detailed mutations and indel statistics. Top 12 genome-wide KSS positions under the three-indel selection rule (Sheet 1) expanded to one row per variant *k*-mer with the same column structure as S10 Table. Sheet 2 provides per-gene indel statistics for gag, pol, and env.
(XLSX)

S12 Table HCMV top-ranking positions: detailed mutations and indel statistics. Top 10 per-gene KSS positions for UL55, UL73, and US28 under the three-indel selection rule (Sheet 1) expanded to one row per variant *k*-mer with the same column structure as S10 Table. Sheet 2 provides per-gene indel statistics.
(XLSX)

## References

[1] Sayers EW, Cavanaugh M, Frisse L, Pruitt KD, Schneider VA, Underwood BA, et al. GenBank 2025 update. Nucleic Acids Res. 2025;53(D1):D56–61. doi: 10.1093/nar/gkae1114 39558184 PMC11701615

[2] Simmonds P, Adams MJ, Benkő M, Breitbart M, Brister JR, Carstens EB, et al. Virus taxonomy in the age of metagenomics. Nat Rev Microbiol. 2017;15(3):161–8. doi: 10.1038/nrmicro.2016.177 28134265

[3] Simmonds P, Aiewsakun P. Virus classification–where do you draw the line? Arch Virol. 2018;163:2037–46. 10.1007/s00705-018-3938-z PMC6096723 30039318

[4] Wood DE, Salzberg SL. Kraken: ultrafast metagenomic sequence classification using exact alignments. Genome Biol. 2014;15(3):R46. doi: 10.1186/gb-2014-15-3-r46 24580807 PMC4053813

[5] Ren J, Ahlgren NA, Lu YY, Fuhrman JA, Sun F. VirFinder: a novel k-mer based tool for identifying viral sequences from assembled metagenomic data. Microbiome. 2017;5(1):69. doi: 10.1186/s40168-017-0283-5 28683828 PMC5501583

[6] Solis-Reyes S, Avino M, Poon A, Kari L. An open-source k-mer based machine learning tool for fast and accurate subtyping of HIV-1 genomes. PLoS One. 2018;13(11):e0206409. doi: 10.1371/journal.pone.0206409 30427878 PMC6235296

[7] Lebatteux D, Remita AM, Diallo AB. Toward an Alignment-Free Method for Feature Extraction and Accurate Classification of Viral Sequences. J Comput Biol. 2019;26(6):519–35. doi: 10.1089/cmb.2018.0239 31050550

[8] Raju RS, Al Nahid A, Chondrow Dev P, Islam R. VirusTaxo: Taxonomic classification of viruses from the genome sequence using k-mer enrichment. Genomics. 2022;114(4):110414. doi: 10.1016/j.ygeno.2022.110414 35718090

[9] Lebatteux D, Soudeyns H, Boucoiran I, Gantt S, Diallo AB. Machine learning-based approach KEVOLVE efficiently identifies SARS-CoV-2 variant-specific genomic signatures. PLoS One. 2024;19(1):e0296627. doi: 10.1371/journal.pone.0296627 38241279 PMC10798494

[10] Zielezinski A, Vinga S, Almeida J, Karlowski WM. Alignment-free sequence comparison: benefits, applications, and tools. Genome Biol. 2017;18(1):186. doi: 10.1186/s13059-017-1319-7 28974235 PMC5627421

[11] Bailey TL, Johnson J, Grant CE, Noble WS. The MEME Suite. Nucleic Acids Res. 2015;43(W1):W39-49. doi: 10.1093/nar/gkv416 25953851 PMC4489269

[12] Bailey TL. STREME: accurate and versatile sequence motif discovery. Bioinformatics. 2021;37(18):2834–40. doi: 10.1093/bioinformatics/btab203 33760053 PMC8479671

[13] Zhang T, Liu Y, Guo X, Zhang X, Zheng X, Zhang M. VISTA: A Tool for Fast Taxonomic Assignment of Viral Genome Sequences. Genomics Proteomics Bioinform. 2024. doi: qzae082 10.1093/gpbjnl/qzae082 PMC12212643 39540753

[14] Alam MNU, Chowdhury UF. Short k-mer abundance profiles yield robust machine learning features and accurate classifiers for RNA viruses. PLoS One. 2020;15(9):e0239381. doi: 10.1371/journal.pone.0239381 32946529 PMC7500682

[15] Saeys Y, Inza I, Larrañaga P. A review of feature selection techniques in bioinformatics. Bioinformatics. 2007;23(19):2507–17. doi: 10.1093/bioinformatics/btm344 17720704

[16] Pimenta-Zanon MH, Kashiwabara AY, Vanzela ALL, Lopes FM. GRAMEP: an alignment-free method based on the maximum entropy principle for identifying SNPs. BMC Bioinform. 2025;26(1):66. doi: 10.1186/s12859-025-06037-z 40000933 PMC11863517

[17] Moeckel C, Mareboina M, Konnaris MA, Chan CSY, Mouratidis I, Montgomery A, et al. A survey of k-mer methods and applications in bioinformatics. Comput Struct Biotechnol J. 2024;23:2289–303. doi: 10.1016/j.csbj.2024.05.025 38840832 PMC11152613

[18] Miyata T, Miyazawa S, Yasunaga T. Two types of amino acid substitutions in protein evolution. J Mol Evol. 1979;12(3):219–36. doi: 10.1007/BF01732340 439147

[19] Zhang Q, Jun S-R, Leuze M, Ussery D, Nookaew I. Viral Phylogenomics Using an Alignment-Free Method: A Three-Step Approach to Determine Optimal Length of k-mer. Sci Rep. 2017;7:40712. doi: 10.1038/srep40712 28102365 PMC5244389

[20] Needleman SB, Wunsch CD. A general method applicable to the search for similarities in the amino acid sequence of two proteins. J Mol Biol. 1970;48(3):443–53. doi: 10.1016/0022-2836(70)90057-4 5420325

[21] Lebatteux D, Soudeyns H, Boucoiran I, Gantt S, Diallo AB. KANALYZER: a method to identify variations of discriminative k-mers in genomic sequences. In: 2022 IEEE International Conference on Bioinformatics and Biomedicine (BIBM). IEEE; 2022. p. 757–62.

[22] Betts MJ, Russell RB. Amino acid properties and consequences of substitutions. Bioinformatics for geneticists. 2003. p. 289–316.

[23] Rudnicki WR, Mroczek T, Cudek P. Amino acid properties conserved in molecular evolution. PLoS One. 2014;9(6):e98983. doi: 10.1371/journal.pone.0098983 24967708 PMC4072533

[24] Das JK, Roy S. A study on non-synonymous mutational patterns in structural proteins of SARS-CoV-2. Genome. 2021;64(7):665–78. doi: 10.1139/gen-2020-0157 33788636

[25] Tomii K, Kanehisa M. Analysis of amino acid indices and mutation matrices for sequence comparison and structure prediction of proteins. Protein Eng. 1996;9(1):27–36. doi: 10.1093/protein/9.1.27 9053899

[26] Dayhoff M, Schwartz R, Orcutt B. 22 a model of evolutionary change in proteins. Atlas of protein sequence and structure. 1978;5:345–52.

[27] Henikoff S, Henikoff JG. Amino acid substitution matrices from protein blocks. Proc Natl Acad Sci U S A. 1992;89(22):10915–9. doi: 10.1073/pnas.89.22.10915 1438297 PMC50453

[28] Kawashima S, Pokarowski P, Pokarowska M, Kolinski A, Katayama T, Kanehisa M. AAindex: amino acid index database, progress report 2008. Nucleic Acids Research. 2007;36(suppl_1):D202-5. 10.1093/nar/gkm998 PMC2238890 17998252

[29] Müller T, Vingron M. Modeling amino acid replacement. J Comput Biol. 2000;7(6):761–76. doi: 10.1089/10665270050514918 11382360

[30] Whelan S, Goldman N. A general empirical model of protein evolution derived from multiple protein families using a maximum-likelihood approach. Mol Biol Evol. 2001;18(5):691–9. doi: 10.1093/oxfordjournals.molbev.a003851 11319253

[31] Nickle DC, Heath L, Jensen MA, Gilbert PB, Mullins JI, Kosakovsky Pond SL. PLoS ONE. 2007;2(6):e503. doi: 10.1371/journal.pone.0000503 PMC1876811 17551583

[32] Dimmic MW, Rest JS, Mindell DP, Goldstein RA. rtREV: an amino acid substitution matrix for inference of retrovirus and reverse transcriptase phylogeny. J Mol Evol. 2002;55(1):65–73. doi: 10.1007/s00239-001-2304-y 12165843

[33] Dang CC, Le QS, Gascuel O, Le VS. FLU, an amino acid substitution model for influenza proteins. BMC Evol Biol. 2010;10:99. doi: 10.1186/1471-2148-10-99 20384985 PMC2873421

[34] Le TK, Vinh LS. FLAVI: An Amino Acid Substitution Model for Flaviviruses. J Mol Evol. 2020;88(5):445–52. doi: 10.1007/s00239-020-09943-3 32356020

[35] Fauchère JL, Charton M, Kier LB, Verloop A, Pliska V. Amino acid side chain parameters for correlation studies in biology and pharmacology. Int J Pept Protein Res. 1988;32(4):269–78. doi: 10.1111/j.1399-3011.1988.tb01261.x 3209351

[36] Grantham R. Amino acid difference formula to help explain protein evolution. Science. 1974;185(4154):862–4. doi: 10.1126/science.185.4154.862 4843792

[37] Krigbaum WR, Komoriya A. Local interactions as a structure determinant for protein molecules: II. Biochim Biophys Acta. 1979;576(1):204–48. 10.1016/0005-2795(79)90498-7 760806

[38] Rose GD, Geselowitz AR, Lesser GJ, Lee RH, Zehfus MH. Hydrophobicity of amino acid residues in globular proteins. Science. 1985;229(4716):834–8. doi: 10.1126/science.4023714 4023714

[39] Zimmerman JM, Eliezer N, Simha R. The characterization of amino acid sequences in proteins by statistical methods. J Theor Biol. 1968;21(2):170–201. doi: 10.1016/0022-5193(68)90069-6 5700434

[40] Miller S, Janin J, Lesk AM, Chothia C. Interior and surface of monomeric proteins. J Mol Biol. 1987;196(3):641–56. doi: 10.1016/0022-2836(87)90038-6 3681970

[41] Hirano A, Kameda T. Aromaphilicity Index of Amino Acids: Molecular Dynamics Simulations of the Protein Binding Affinity for Carbon Nanomaterials. ACS Appl Nano Mater. 2021;4(3):2486–95. doi: 10.1021/acsanm.0c03047

[42] Xia X, Xia X. Protein substitution model and evolutionary distance. Bioinformatics and the Cell: Modern Computational Approaches in Genomics, Proteomics and Transcriptomics. 2018. p. 315–26.

[43] Ashburner M, Ball CA, Blake JA, Botstein D, Butler H, Cherry JM, et al. Gene Ontology: tool for the unification of biology. Nat Genet. 2000;25(1):25–9. doi: 10.1038/75556 PMC3037419 10802651

[44] Apetrei C, Hahn B, Rambaut A, Wolinsky S, Brister JR, Keele B, et al. HIV sequence compendium 2021. Theor Biol Biophys Group. 2021;699:700.

[45] Rambaut A, Holmes EC, O’Toole Á, Hill V, McCrone JT, Ruis C, et al. A dynamic nomenclature proposal for SARS-CoV-2 lineages to assist genomic epidemiology. Nat Microbiol. 2020;5(11):1403–7. doi: 10.1038/s41564-020-0770-5 32669681 PMC7610519

[46] Aksamentov I, Roemer C, Hodcroft E, Neher R. Nextclade: clade assignment, mutation calling and quality control for viral genomes. JOSS. 2021;6(67):3773. doi: 10.21105/joss.03773

[47] Arav-Boger R, Willoughby RE, Pass RF, Zong J-C, Jang W-J, Alcendor D, et al. Polymorphisms of the cytomegalovirus (CMV)-encoded tumor necrosis factor-alpha and beta-chemokine receptors in congenital CMV disease. J Infect Dis. 2002;186(8):1057–64. doi: 10.1086/344238 12355354

[48] Chou SW, Dennison KM. Analysis of interstrain variation in cytomegalovirus glycoprotein B sequences encoding neutralization-related epitopes. J Infect Dis. 1991;163(6):1229–34. doi: 10.1093/infdis/163.6.1229 1709960

[49] Pignatelli S, Dal Monte P, Landini MP. gpUL73 (gN) genomic variants of human cytomegalovirus isolates are clustered into four distinct genotypes. J Gen Virol. 2001;82(Pt 11):2777–84. doi: 10.1099/0022-1317-82-11-2777 11602789

[50] Pearson K. X. On the criterion that a given system of deviations from the probable in the case of a correlated system of variables is such that it can be reasonably supposed to have arisen from random sampling. Lond Edinb Dubl Phil Mag J Sci. 1900;50(302):157–75. doi: 10.1080/14786440009463897

[51] Cover TM. Elements of information theory. John Wiley & Sons; 1999.

[52] Breslow NE. Statistics in epidemiology: the case-control study. J Am Stat Assoc. 1996;91(433):14–28. doi: 10.1080/01621459.1996.10476660 12155399

[53] Cramér H. Mathematical methods of statistics. vol. 9. Princeton University Press; 1999.

[54] Fisher RA. Statistical methods for research workers. In: Breakthroughs in statistics: Methodology and distribution. Springer; 1970. p. 66–70.

[55] Topsøe F. Some inequalities for information divergence and related measures of discrimination. IEEE Trans Inform Theory. 2000;46(4):1602–9.

[56] Lebatteux D, Diallo AB. Combining a genetic algorithm and ensemble method to improve the classification of viruses. In: 2021 IEEE International Conference on Bioinformatics and Biomedicine (BIBM). IEEE; 2021. p. 688–93.

[57] Wade KE, Chen L, Deng C, Zhou G, Hu P. Investigating alignment-free machine learning methods for HIV-1 subtype classification. Bioinform Adv. 2024;4(1):vbae108. doi: 10.1093/bioadv/vbae108 39228995 PMC11371153

[58] Nadeau C, Bengio Y. Inference for the Generalization Error. Mach Learn. 2003;52(3):239–81. doi: 10.1023/a:1024068626366

[59] Holm S. A simple sequentially rejective multiple test procedure. Scand J Stat. 1979;6(2):65–70.

[60] Lakens D. Equivalence Tests: A Practical Primer for t Tests, Correlations, and Meta-Analyses. Soc Psychol Personal Sci. 2017;8(4):355–62. doi: 10.1177/1948550617697177 28736600 PMC5502906

[61] Demšar J. Statistical comparisons of classifiers over multiple data sets. J Mach Learn Res. 2006;7:1–30.

[62] Feng Y, Yi J, Yang L, Wang Y, Wen J, Zhao W, et al. COV2Var, a function annotation database of SARS-CoV-2 genetic variation. Nucleic Acids Res. 2024;52(D1):D701–13. doi: 10.1093/nar/gkad958 37897356 PMC10767816

[63] Feng DF, Johnson MS, Doolittle RF. Aligning amino acid sequences: comparison of commonly used methods. J Mol Evol. 1984;21(2):112–25. doi: 10.1007/BF02100085 6100188

[64] Schneider M, Lane L, Boutet E, Lieberherr D, Tognolli M, Bougueleret L, et al. The UniProtKB/Swiss-Prot knowledgebase and its Plant Proteome Annotation Program. J Proteomics. 2009;72(3):567–73. doi: 10.1016/j.jprot.2008.11.010 19084081 PMC2689360

[65] Boutet E, Lieberherr D, Tognolli M, Schneider M, Bansal P, Bridge AJ, et al. UniProtKB/Swiss-Prot, the manually annotated section of the UniProt KnowledgeBase: how to use the entry view. In: Plant bioinformatics: methods and protocols. Springer; 2016. p. 23–54. 10.1007/978-1-4939-3167-5_2 26519399

[66] Chen Y, Li Y, Zhou Q, Cong Z, Lin S, Yan J, et al. A homotrimeric GPCR architecture of the human cytomegalovirus revealed by cryo-EM. Cell Discov. 2024;10(1):52. doi: 10.1038/s41421-024-00684-x 38750025 PMC11096299

[67] Medica S, Diggins NL, Denton M, Turner RL, Pung LJ, Mayo AT, et al. Human cytomegalovirus UL78 is a nuclear-localized GPCR necessary for efficient reactivation from latent infection in CD34+ hematopoietic progenitor cells. J Virol. 2025;99(11):e0140225. doi: 10.1128/jvi.01402-25 41060092 PMC12645970

[68] McCarthy KR, Rennick LJ, Nambulli S, Robinson-McCarthy LR, Bain WG, Haidar G, et al. Recurrent deletions in the SARS-CoV-2 spike glycoprotein drive antibody escape. Science. 2021;371(6534):1139–42. doi: 10.1126/science.abf6950 33536258 PMC7971772

[69] Harvey WT, Carabelli AM, Jackson B, Gupta RK, Thomson EC, Harrison EM, et al. SARS-CoV-2 variants, spike mutations and immune escape. Nat Rev Microbiol. 2021;19(7):409–24. doi: 10.1038/s41579-021-00573-0 34075212 PMC8167834

[70] McCallum M, De Marco A, Lempp FA, Tortorici MA, Pinto D, Walls AC, et al. N-terminal domain antigenic mapping reveals a site of vulnerability for SARS-CoV-2. Cell. 2021;184(9):2332–47.e16. doi: 10.1016/j.cell.2021.03.028 33761326 PMC7962585

[71] Carabelli AM, Peacock TP, Thorne LG, Harvey WT, Hughes J, COVID-19 Genomics UK Consortium, et al. SARS-CoV-2 variant biology: immune escape, transmission and fitness. Nat Rev Microbiol. 2023;21(3):162–77. doi: 10.1038/s41579-022-00841-7 36653446 PMC9847462

[72] Liu L, Iketani S, Guo Y, Chan JF-W, Wang M, Liu L, et al. Striking antibody evasion manifested by the Omicron variant of SARS-CoV-2. Nature. 2022;602(7898):676–81. doi: 10.1038/s41586-021-04388-0 35016198

[73] Iketani S, Liu L, Guo Y, Liu L, Chan JF-W, Huang Y, et al. Antibody evasion properties of SARS-CoV-2 Omicron sublineages. Nature. 2022;604(7906):553–6. doi: 10.1038/s41586-022-04594-4 35240676 PMC9021018

[74] Elko EA, Mead HL, Nelson GA, Zaia JA, Ladner JT, Altin JA. Recurrent SARS-CoV-2 mutations at Spike D796 evade antibodies from pre-Omicron convalescent and vaccinated subjects. Microbiol Spectr. 2024;12(2):e0329123. doi: 10.1128/spectrum.03291-23 38189279 PMC10871546

[75] Wu H, Xing N, Meng K, Fu B, Xue W, Dong P, et al. Nucleocapsid mutations R203K/G204R increase the infectivity, fitness, and virulence of SARS-CoV-2. Cell Host Microbe. 2021;29(12):1788-1801.e6. doi: 10.1016/j.chom.2021.11.005 34822776 PMC8590493

[76] Voss C, Esmail S, Liu X, Knauer MJ, Ackloo S, Kaneko T, et al. Epitope-specific antibody responses differentiate COVID-19 outcomes and variants of concern. JCI Insight. 2021;6(13):e148855. doi: 10.1172/jci.insight.148855 34081630 PMC8410046

[77] Hossain A, Akter S, Rashid AA, Khair S, Alam ASMRU. Unique mutations in SARS-CoV-2 Omicron subvariants’ non-spike proteins: Potential impacts on viral pathogenesis and host immune evasion. Microb Pathog. 2022;170:105699. doi: 10.1016/j.micpath.2022.105699 35944840 PMC9356572

[78] Leiherer A, Ludwig C, Wagner R. Uncoupling human immunodeficiency virus type 1 Gag and Pol reading frames: role of the transframe protein p6* in viral replication. J Virol. 2009;83(14):7210–20. doi: 10.1128/JVI.02603-08 19403679 PMC2704792

[79] Wensing AM, Calvez V, Ceccherini-Silberstein F, Charpentier C, Günthard HF, Paredes R, et al. 2022 update of the drug resistance mutations in HIV-1. Top Antivir Med. 2022;30(4):559–74. 36375130 PMC9681141

[80] Sarafianos SG, Marchand B, Das K, Himmel DM, Parniak MA, Hughes SH, et al. Structure and function of HIV-1 reverse transcriptase: molecular mechanisms of polymerization and inhibition. J Mol Biol. 2009;385(3):693–713. doi: 10.1016/j.jmb.2008.10.071 19022262 PMC2881421

[81] Svicher V, Sing T, Santoro MM, Forbici F, Rodríguez-Barrios F, Bertoli A, et al. Involvement of novel human immunodeficiency virus type 1 reverse transcriptase mutations in the regulation of resistance to nucleoside inhibitors. J Virol. 2006;80(14):7186–98. doi: 10.1128/JVI.02084-05 16809324 PMC1489024

[82] Saracino A, Monno L, Scudeller L, Cibelli DC, Tartaglia A, Punzi G, et al. Impact of unreported HIV-1 reverse transcriptase mutations on phenotypic resistance to nucleoside and non-nucleoside inhibitors. J Med Virol. 2006;78(1):9–17. doi: 10.1002/jmv.20500 16299731

[83] Roquebert B, Marcelin AG. Role of HIV-1 reverse transcriptase RNase H domain in resistance to nucleoside analogues. Virologie. 2008;12(3):183–92. doi: 10.1684/vir.2008.0157

[84] Engelman A, Liu Y, Chen H, Farzan M, Dyda F. Structure-based mutagenesis of the catalytic domain of human immunodeficiency virus type 1 integrase. J Virol. 1997;71(5):3507–14. doi: 10.1128/JVI.71.5.3507-3514.1997 9094622 PMC191497

[85] Mazumder A, Neamati N, Pilon AA, Sunder S, Pommier Y. Chemical trapping of ternary complexes of human immunodeficiency virus type 1 integrase, divalent metal, and DNA substrates containing an abasic site. Implications for the role of lysine 136 in DNA binding. J Biol Chem. 1996;271(44):27330–8. doi: 10.1074/jbc.271.44.27330 8910309

[86] van Hal SJ, Herring B, Deris Z, Wang B, Saksena NK, Dwyer DE. HIV-1 integrase polymorphisms are associated with prior antiretroviral drug exposure. Retrovirology. 2009;6:12. doi: 10.1186/1742-4690-6-12 19203393 PMC2649883

[87] Davey NE, Satagopam VP, Santiago-Mozos S, Villacorta-Martin C, Bharat TAM, Schneider R, et al. The HIV mutation browser: a resource for human immunodeficiency virus mutagenesis and polymorphism data. PLoS Comput Biol. 2014;10(12):e1003951. doi: 10.1371/journal.pcbi.1003951 25474213 PMC4256008

[88] Brandano L, Stevenson M. A highly conserved residue in the C-terminal helix of HIV-1 matrix is required for envelope incorporation into virus particles. J Virol. 2012;86(4):2347–59. doi: 10.1128/JVI.06047-11 22156517 PMC3302379

[89] Brockman MA, Brumme ZL, Brumme CJ, Miura T, Sela J, Rosato PC, et al. Early selection in Gag by protective HLA alleles contributes to reduced HIV-1 replication capacity that may be largely compensated for in chronic infection. J Virol. 2010;84(22):11937–49. doi: 10.1128/JVI.01086-10 20810731 PMC2977869

[90] Stuchell MD, Garrus JE, Müller B, Stray KM, Ghaffarian S, McKinnon R, et al. The human endosomal sorting complex required for transport (ESCRT-I) and its role in HIV-1 budding. J Biol Chem. 2004;279(34):36059–71. doi: 10.1074/jbc.M405226200 15218037

[91] Wanaguru M, Bishop KN. HIV-1 Gag Recruits Oligomeric Vpr via Two Binding Sites in p6, but Both Mature p6 and Vpr Are Rapidly Lost upon Target Cell Entry. J Virol. 2021;95(17):e00554-21. doi: 10.1128/JVI.00554-21 34106747 PMC8354229

[92] Flys T, Marlowe N, Hackett J, Parkin N, Schumaker M, Holzmayer V, et al. Analysis of PTAP duplications in the gag p6 region of subtype C HIV type 1. AIDS Res Hum Retroviruses. 2005;21(8):739–41. doi: 10.1089/aid.2005.21.739 16131315

[93] van Domselaar R, Njenda DT, Rao R, Sönnerborg A, Singh K, Neogi U. HIV-1 Subtype C with PYxE Insertion Has Enhanced Binding of Gag-p6 to Host Cell Protein ALIX and Increased Replication Fitness. J Virol. 2019;93(9):e00077-19. doi: 10.1128/JVI.00077-19 30760577 PMC6475790

[94] Kwong PD, Wyatt R, Robinson J, Sweet RW, Sodroski J, Hendrickson WA. Structure of an HIV gp120 envelope glycoprotein in complex with the CD4 receptor and a neutralizing human antibody. Nature. 1998;393(6686):648–59. doi: 10.1038/31405 9641677 PMC5629912

[95] Pignatelli S, Lazzarotto T, Gatto MR, Dal Monte P, Landini MP, Faldella G, et al. Cytomegalovirus gN genotypes distribution among congenitally infected newborns and their relationship with symptoms at birth and sequelae. Clin Infect Dis. 2010;51(1):33–41. doi: 10.1086/653423 20504230

[96] Waters S, Agostino M, Lee S, Ariyanto I, Kresoje N, Leary S, et al. Sequencing Directly from Clinical Specimens Reveals Genetic Variations in HCMV-Encoded Chemokine Receptor US28 That May Influence Antibody Levels and Interactions with Human Chemokines. Microbiol Spectr. 2021;9(2):e0002021. doi: 10.1128/Spectrum.00020-21 34704798 PMC8549752

[97] Stropes MP, Schneider OD, Zagorski WA, Miller JLC, Miller WE. The carboxy-terminal tail of human cytomegalovirus (HCMV) US28 regulates both chemokine-independent and chemokine-dependent signaling in HCMV-infected cells. J Virol. 2009;83(19):10016–27. doi: 10.1128/JVI.00354-09 19605482 PMC2748033

[98] Bloom JD, Neher RA. Fitness effects of mutations to SARS-CoV-2 proteins. Virus Evolution. 2023;9(2):vead055. 10.1093/ve/vead055 PMC10506532 37727875

[99] Mahmoudabadi G, Phillips R. A comprehensive and quantitative exploration of thousands of viral genomes. Elife. 2018;7:e31955. doi: 10.7554/eLife.31955 29624169 PMC5908442

[100] Haynes WA, Tomczak A, Khatri P. Gene annotation bias impedes biomedical research. Sci Rep. 2018;8(1):1362. doi: 10.1038/s41598-018-19333-x 29358745 PMC5778030

[101] Stoeger T, Gerlach M, Morimoto RI, Nunes Amaral LA. Large-scale investigation of the reasons why potentially important genes are ignored. PLOS Biol. 2018;16(9):e2006643. 10.1371/journal.pbio.2006643 PMC6143198 30226837

[102] Kustatscher G, Collins T, Gingras A-C, Guo T, Hermjakob H, Ideker T, et al. Understudied proteins: opportunities and challenges for functional proteomics. Nat Methods. 2022;19(7):774–9. doi: 10.1038/s41592-022-01454-x 35534633

[103] Altschul SF. Amino acid substitution matrices from an information theoretic perspective. J Mol Biol. 1991;219(3):555–65. doi: 10.1016/0022-2836(91)90193-a 2051488 PMC7130686

[104] Sneath PH. Relations between chemical structure and biological activity in peptides. J Theor Biol. 1966;12(2):157–95. doi: 10.1016/0022-5193(66)90112-3 4291386

